# 2D-3D reconstruction of the proximal femur from DXA scans: Evaluation of the 3D-Shaper software

**DOI:** 10.3389/fbioe.2023.1111020

**Published:** 2023-03-01

**Authors:** Alice Dudle, Yvan Gugler, Michael Pretterklieber, Serge Ferrari, Kurt Lippuner, Philippe Zysset

**Affiliations:** ^1^ ARTORG Center for Biomedical Engineering Research, University of Bern, Bern, Switzerland; ^2^ Division of Anatomy, Gottfried Schatz Research Center, Medical University of Graz, Graz, Austria; ^3^ Division of Anatomy, Center for Anatomy and Cell Biology, Medical University of Vienna, Vienna, Austria; ^4^ Division of Bone Diseases, Geneva University Hospitals (HUG), Geneva, Switzerland; ^5^ Department of Osteoporosis, Inselspital, Bern University Hospital, University of Bern, Bern, Switzerland

**Keywords:** 3D-Shaper, DXA, QCT, FE, bone strength, femur, BMD, 2D-3D reconstruction

## Abstract

**Introduction:** Osteoporosis is currently diagnosed based on areal bone mineral density (aBMD) computed from 2D DXA scans. However, aBMD is a limited surrogate for femoral strength since it does not account for 3D bone geometry and density distribution. QCT scans combined with finite element (FE) analysis can deliver improved femoral strength predictions. However, non-negligible radiation dose and high costs prevent a systematic usage of this technique for screening purposes. As an alternative, the 3D-Shaper software (3D-Shaper Medical, Spain) reconstructs the 3D shape and density distribution of the femur from 2D DXA scans. This approach could deliver a more accurate estimation of femoral strength than aBMD by using FE analysis on the reconstructed 3D DXA.

**Methods:** Here we present the first independent evaluation of the software, using a dataset of 77 *ex vivo* femora. We extend a prior evaluation by including the density distribution differences, the spatial correlation of density values and an FE analysis. Yet, cortical thickness is left out of this evaluation, since the cortex is not resolved in our FE models.

**Results:** We found an average surface distance of 1.16 mm between 3D DXA and QCT images, which shows a good reconstruction of the bone geometry. Although BMD values obtained from 3D DXA and QCT correlated well (*r* ^2^ = 0.92), the 3D DXA BMD were systematically lower. The average BMD difference amounted to 64 mg/cm^3^, more than one-third of the 3D DXA BMD. Furthermore, the low correlation (*r* ^2^ = 0.48) between density values of both images indicates a limited reconstruction of the 3D density distribution. FE results were in good agreement between QCT and 3D DXA images, with a high coefficient of determination (*r* ^2^ = 0.88). However, this correlation was not statistically different from a direct prediction by aBMD. Moreover, we found differences in the fracture patterns between the two image types. QCT-based FE analysis resulted mostly in femoral neck fractures and 3D DXA-based FE in subcapital or pertrochanteric fractures.

**Discussion:** In conclusion, 3D-Shaper generates an altered BMD distribution compared to QCT but, after careful density calibration, shows an interesting potential for deriving a standardized femoral strength from a DXA scan.

## 1 Introduction

Osteoporosis is characterized by a loss of bone mass which induces a decrease in bone strength. The disease is generally silent until a fragility fracture occurs, often resulting in a loss of independence and quality of life for the patient (Kanis et al., 2021). The diagnosis of osteoporosis is currently defined as a low T-score, which is computed from the areal bone mineral density (aBMD) extracted from 2D dual-energy X-ray absorptiometry (DXA) images at the lumbar spine and the proximal femur. If a quantitative computer tomography (QCT) scan including the proximal part of the femur is available, an equivalent aBMD score can be computed by projecting the image onto a 2D plane (Khoo et al., 2009).

However, aBMD is a limited surrogate for bone strength since it does not account for bone geometry and spatial distribution of bone density. Moreover, fracture risk depends on additional factors. The FRAX tool (McCloskey et al., 2012) relies on different clinical risk factors (e.g., BMI, current smoking status) in combination with or without aBMD to compute a 10-year fracture risk. More “mechanistic” models of hip fracture risk (Schechner et al., 2010; Bhattacharya et al., 2019) may further improve fracture risk estimation. However, these “mechanistic” models require an estimate of femoral strength in dimension of force (N) rather than an aBMD value. Increasing evidence shows that femoral strength can be predicted precisely by finite element (FE) analysis of QCT scans (Engelke et al., 2015; Bouxsein et al., 2020). Thus, instead of projecting QCT images to get DXA-equivalent scores, it is meaningful to reconstruct the DXA images into 3D models of the femur and compute femoral strength with FE analysis (Thevenot et al., 2014; Väänänen et al., 2015; Grassi et al., 2017; Ruiz Wills et al., 2019; Grassi et al., 2021).

Most reconstruction methods so far use statistical models of shape and density (Reyneke et al., 2019) to recover a 3D image of the bone from a single (Langton et al., 2009; Whitmarsh et al., 2011) or a few (Zheng et al., 2009; Ehlke et al., 2013) 2D projection(s). The commercial software 3D-Shaper (3D-Shaper Medical, Spain) provides the 3D reconstruction of the proximal part of the femur, as well as other metrics related to bone strength, based on a single DXA scan (Humbert et al., 2017). In brief, the software uses a statistical model of shape and density derived from a dataset of QCT scans of the femur. Iteratively, the femur model is repositioned and deformed, before being projected and compared to the original 2D DXA scan. The model instance producing the projection closest to the DXA image is selected as reconstruction. Lately, the 3D-Shaper software has experienced a growing interest in the clinical community. Indeed, 3D-Shaper has recently been used to evaluate different treatment strategies for conditions of skeletal fragility, where especially differential effects of the treatment on cortical and trabecular bone densities were examined (Lewiecki et al., 2022; Winzenrieth et al., 2022). In another initiative, the effect of exercise on femoral neck strength was evaluated using 3D DXA-based FE analysis (O’Rourke et al., 2022).

However, so far, the method’s performance has only been evaluated by the team who developed the reconstruction algorithm (Whitmarsh et al., 2011; Humbert et al., 2017), except for the repeatability, which was evaluated by a separate team (O’Rourke et al., 2021). The present work aims to provide both an independent verification and an extension of the initial evaluation, while a separate study will assess the repeatability. We intend to assess if 3D DXA reconstructed with 3D-Shaper is an adequate surrogate for QCT and if it has the potential to improve fragility fracture risk estimation. For that purpose, we use a new and independent dataset of anatomic specimens of non-embalmed human femora. In addition to the metrics presented by Humbert et al. (2017), such as surface distance and bone mineral density (BMD), we analyze the distribution of the density differences, compute the spatial correlation of BMD values and conduct an FE analysis on both QCT and 3D DXA images. Differences in scalar strength outcomes as well as scalar fields describing the damage of bone tissue are analyzed to get a better picture of the differences between the two methods. On the other hand, we decided to leave cortical thickness out of the analysis. Indeed, cortical thickness is a measurement with high variability since it depends on the resolution of the image and because the definition of the inner cortical surface remains controversial (Zebaze and Seeman, 2015). In addition, cortical thickness as a measure is not relevant for the FE analysis since the cortex is not resolved in our FE models.

To sum up, the aim of our analysis is to shed light on the limitations and potential of 3D DXA for prediction of femoral strength and possibly other clinical applications of the technique.

## 2 Materials and methods

### 2.1 Dataset

Eighty-three human femora were collected from forty-two donors by the Division of Anatomy of the Medical University of Vienna. The human bone material was collected with prior consent of the donor and no payment was received. Six femora were excluded due to pre-existing neck fractures or to the presence of large air bubbles (visible in the CT scan), resulting in a dataset of seventy-seven femora from forty-one donors. The dataset is described in Table 1. One donor had unknown sex and age. Osteopenia and osteoporosis were defined based on the DXA-based neck T-score. For pairs of femora from the same donor, the worst of both T-scores was used to classify the donor, resulting in a larger proportion of osteoporotic donors than femora. The study focuses on the proximal part of the femur, which is called proximal femur in the following text for simplicity.

**TABLE 1:** Description of donor demographics.

	Female	Male	Gender unknown	Mean age (range)	Normal BMD	Osteopenia	Osteoporosis
Donors	21	19	1	81 ± 9 (57–96)	3	8	30
Femora	39	36	2	80 ± 9 (57–96)	7	18	52

QCT scans were realized in October 2017 (17 femora, subset I) and in March 2019 (66 femora, subset II) at the Institute of Forensic Medicine in Bern, on a CT scanner (SOMATOM Definition AS, Siemens, Germany) with a calibration phantom (BDC 700 mm with six inserts, QRM, Germany). The CT images were cropped approximately 1 cm distal to the lesser trochanter and resampled to an isotropic resolution of 1 mm (the same resolution as the reconstructed DXA images) for the following analysis.

**FIGURE 1:**
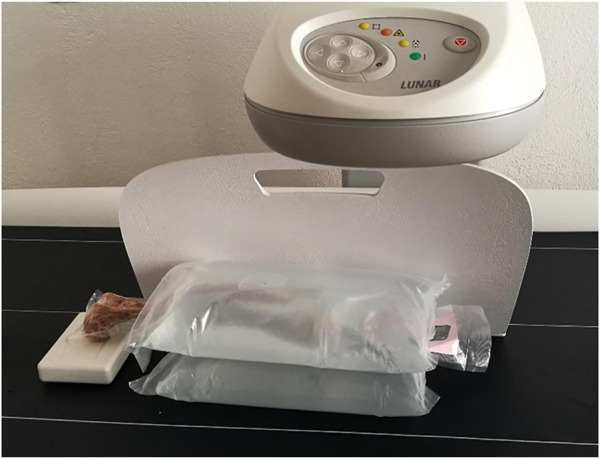
DXA scanning setup: femur between two water bags.

DXA scans were taken in February 2020 on a regularly maintained and calibrated Lunar iDXA (GE, United States) at a satellite site of the Department of Osteoporosis, Inselspital Bern. To replace soft tissues, the femora were placed between two plastic bags filled with water, as illustrated in Figure 1, resulting in a total setup height of approximately 15 cm. A water bath of 15 cm depth is frequently encountered in literature to replace soft tissues in DXA scans (Boehm et al., 2008; Holzer et al., 2009; Roberts et al., 2010). The condyles were placed on a flat surface and the resulting angle of the femoral head was maintained while placing the bags, to reproduce the anatomical position of the femur.

### 2.2 CT calibration

The position of the six phantom inserts in the CT image was detected automatically and the average Hounsfield unit (HU) value in the inserts was computed for each slice. The HU values in the inserts showed considerable fluctuations over the scan length. This is probably due to the current modulation used to optimize the radiation dose according to the mass present in each slice. Therefore, the images were calibrated slice per slice, using a moving average of the HU values in the inserts over five slices to remove noise. In five images from subset I and six images from subset II, the calibration phantom was not visible on the whole scan length. For these scans, we computed the relation between the HU values in the inserts and the average HU value in the slice, used as a surrogate for the mass present in each slice. With the help of that relation, the phantom values were extrapolated to the remaining slices. The images were then calibrated as described previously.

### 2.3 Segmentation

To compare both image types, the bone had to be isolated from the image background. For the 3D DXA, we used the cortical surface mesh available in the 3D-Shaper output to define the bone contour. The 3D image and the mesh were overlaid and all image voxels inside the mesh were labeled as bone, while the others were labeled as background. For the QCT images, segmentation masks had to be defined. However, the presence of tissue around the bone reduced the contrast of the bone surface and prevented the use of a thresholding method. Instead, we chose a deep learning approach, starting by segmenting a dozen of images manually. A small U-Net-inspired 3D neural network (Ronneberger et al., 2015) was then trained with these image-segmentation pairs. More details on the network architecture can be found in the Supplementary Material. After training, the neural network was used to predict the segmentation for another group of scans. The predictions were corrected manually and added to the training set. The neural network was then re-trained and used again to predict the segmentation of a further group of scans. The procedure was repeated until segmentation masks were available for all CT images.

### 2.4 DXA reconstruction

DXA scans were reconstructed with the 3D-Shaper software (version 2.10, 3D-Shaper Medical, Barcelona, Spain) following the provided guidelines. First, the DXA image was imported in the software. Three landmarks are required for the reconstruction and are automatically placed by the software. This automatic positioning failed in our case, probably because of the missing pelvis bone, and had to be corrected manually. After positioning the landmarks, a 3D image of the proximal femur was reconstructed iteratively by the software, as described in the introduction. Finally, the software saved the output as images and meshes. Among these output files, we used the 3D image containing the BMD values in the proximal femur, the segmentation mask defining the femoral neck, greater and lesser trochanter regions, and the mesh describing the external cortical surface.

### 2.5 Registration CT-3D DXA

Since the CT and the DXA scans were not performed in the same position, both images had to be aligned before they could be compared. All registrations were performed using the SimpleElastix Python library (version 0.10.0), based on the open-source software Elastix (Klein et al., 2010). To align the CT and the 3D DXA images, we started by computing the registration between both segmentation masks, without taking the BMD values into account. This step allowed to obtain a robust first guess for the alignment by avoiding possible local minima due to the BMD values. As a second step, the alignment was refined according to the BMD distributions. In both cases, the registration transform was rigid, allowing only rotation and translation, so the shapes were not deformed. A pyramidal scheme with four different resolutions was adopted, starting the registration process with a downsampled image and iteratively increasing the resolution to refine the alignment. Mutual information (MI) (Unser and Thevenaz, 2000) was used as an optimization metric. A B-Spline interpolator of the third order was used to compute the BMD values after the transformation (Unser, 1999).

### 2.6 Regions of interest

**FIGURE 2:**
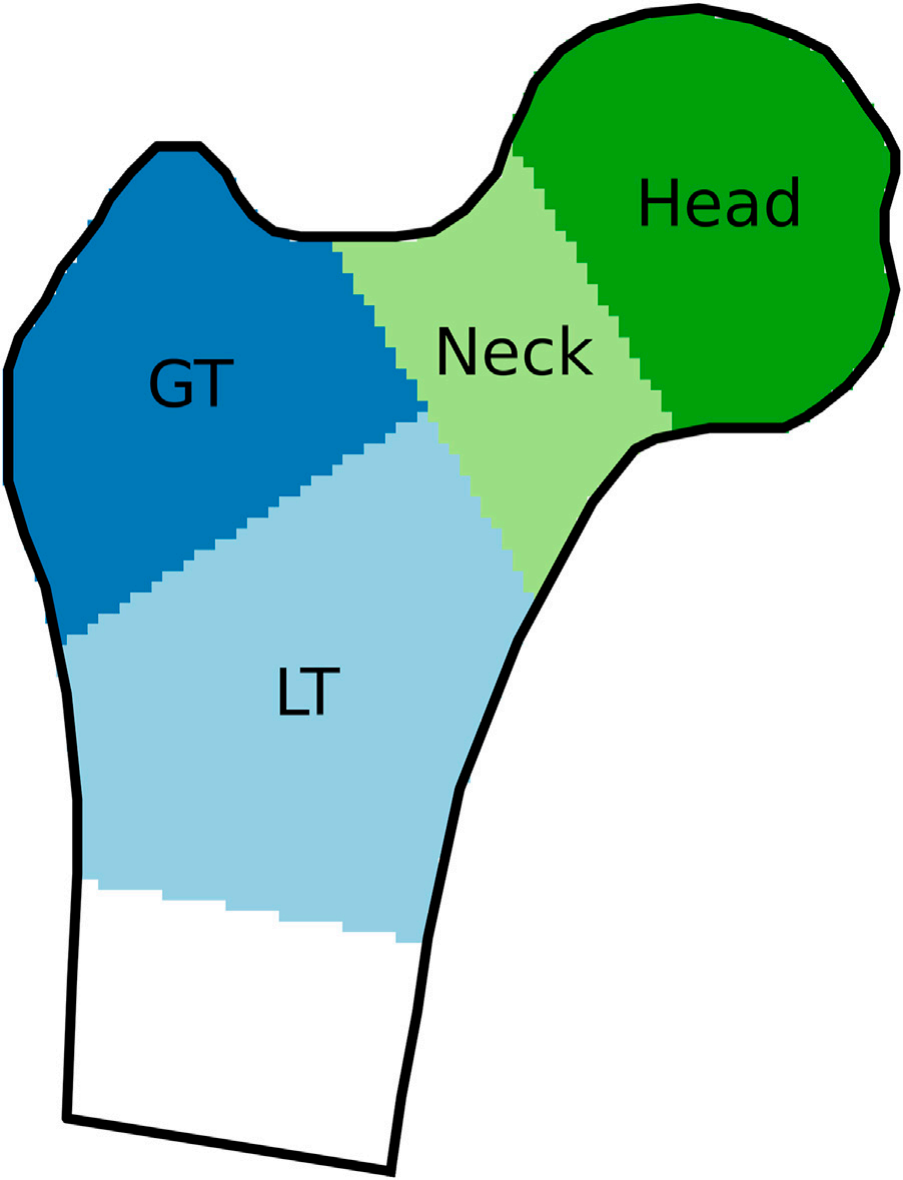
Regions of interest, indicated on a coronal cross-section of a proximal femur. GT and LT stand for greater and lesser trochanter, respectively.

The results were evaluated for the femoral neck, the regions of the greater and lesser trochanter (GT and LT, named trochanter and shaft in Humbert et al., 2017), the combination of these three regions, as well as for the femoral head, as shown in Figure 2. The femoral head was evaluated separately because the corresponding region in the DXA image is usually superimposed with the acetabulum and thus not considered in the reconstruction. These regions were based on the labels present in the 3D-Shaper output. For the CT image, the region boundaries were adapted to fit the bone surface, without moving the planes separating the regions.

### 2.7 Geometrical comparison

First, the volume of each region of interest was computed for the 3D DXA and the CT images. Next, we determined the average and maximal distances between the bone surfaces for each pair of images. The exterior cortical mesh of the 3D DXA was available in the 3D-Shaper output, with anatomically corresponding nodes for all reconstructions. For every node of the 3D DXA mesh, we searched for the nearest point on the registered CT segmentation surface and measured the unsigned distance between them. The average and maximal distances were computed for each region of interest and averaged over all femurs. We also analyzed the spatial distribution of the surface distances. For each node, the average distance over all femora was computed and projected onto the surface mesh of a femur representative of the dataset according to a distance metric presented in Chandran et al. (2017), and called “standard femur” in the following.

### 2.8 BMD comparison

Bone mineral density (BMD) and content (BMC) values of the 3D DXA and CT images were averaged for each region of interest. The voxel-voxel BMD correlation between the CT and 3D DXA images was then computed in the region where the bones overlap. In addition, we analyzed the distribution of the density differences between the CT image and the 3D DXA reconstruction. For that purpose, the images were first downsampled to an isotropic resolution of 3 mm to avoid local density outliers. All 3D DXA reconstructions were then registered non-rigidly to the standard femur. For each femur, the resulting transformation was also applied to the corresponding CT image. After registration, all images were cropped at the same height along the shaft, to keep only the area common to all images. Finally, we computed the BMD difference for all pairs of CT and 3D DXA images and averaged the results for each voxel.

### 2.9 FE analysis

Isotropically resampled, masked, and calibrated CT images and 3D DXA images were used as input for an FE modeling pipeline. The fully automated pipeline created voxel-based FE models and ran non-linear quasi-static FE analyses. The pipeline was implemented in Python and developed and adjusted to enable an integration in the clinical workflow. This required short processing times and a minimum of user input. All code is on GitHub and can be made available upon request.

#### 2.9.1 Implicit coordinate system of the proximal part of the femur

**FIGURE 3:**
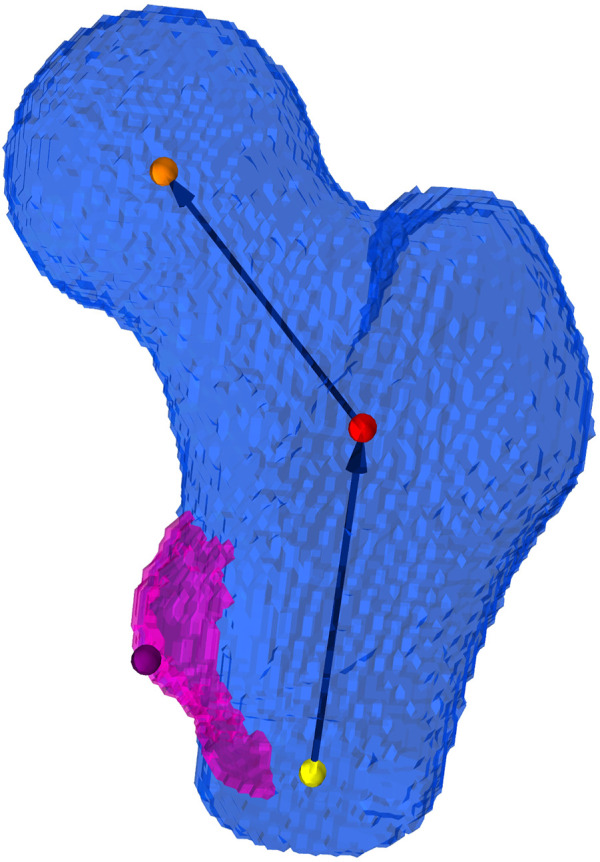
Implicit coordinate system of the proximal femur. The coordinate system consists of an estimate of the femoral neck axis and the proximal shaft axis of the femur, which intersect at a closest point (red dot) defined from two generally not intersecting initial guesses. The orange dot depicts the center of the femoral head, the yellow depicts the distal point used for the definition of the femoral shaft axis, and the purple dot is the peak of the lesser trochanter.

To transform the extracted femoral geometry to the desired experimental position, an implicit coordinate system (Figure 3) of the proximal part of the femur, inspired by Chandran et al. (2017), was established. The coordinate system consisted in an axis characterizing the neck and another axis characterizing the shaft of the proximal femur. An iterative procedure was adopted to obtain two intersecting axes from non-intersecting initial guesses. Thus, the definition could be reduced to three points: one in the center of the femoral head, a second one at the intersection point of the two axes and a third point at the distal end of the proximal femur. The location of the section used to make an initial guess for the shaft axis was placed at a clearly defined height with respect to the lesser trochanter (LT), which was detected along with the two axes. This approach was chosen in order to reduce the influence of the curvature of the femoral shaft and the location of the distal cut plane on the estimated shaft axis. A sphere fit to the femoral head allowed to estimate the radius and center of the femoral head. For a more detailed description of the procedure used to establish the implicit coordinate system, the reader is referred to Supplementary Section S1.2.1.

#### 2.9.2 FE model

**FIGURE 4:**
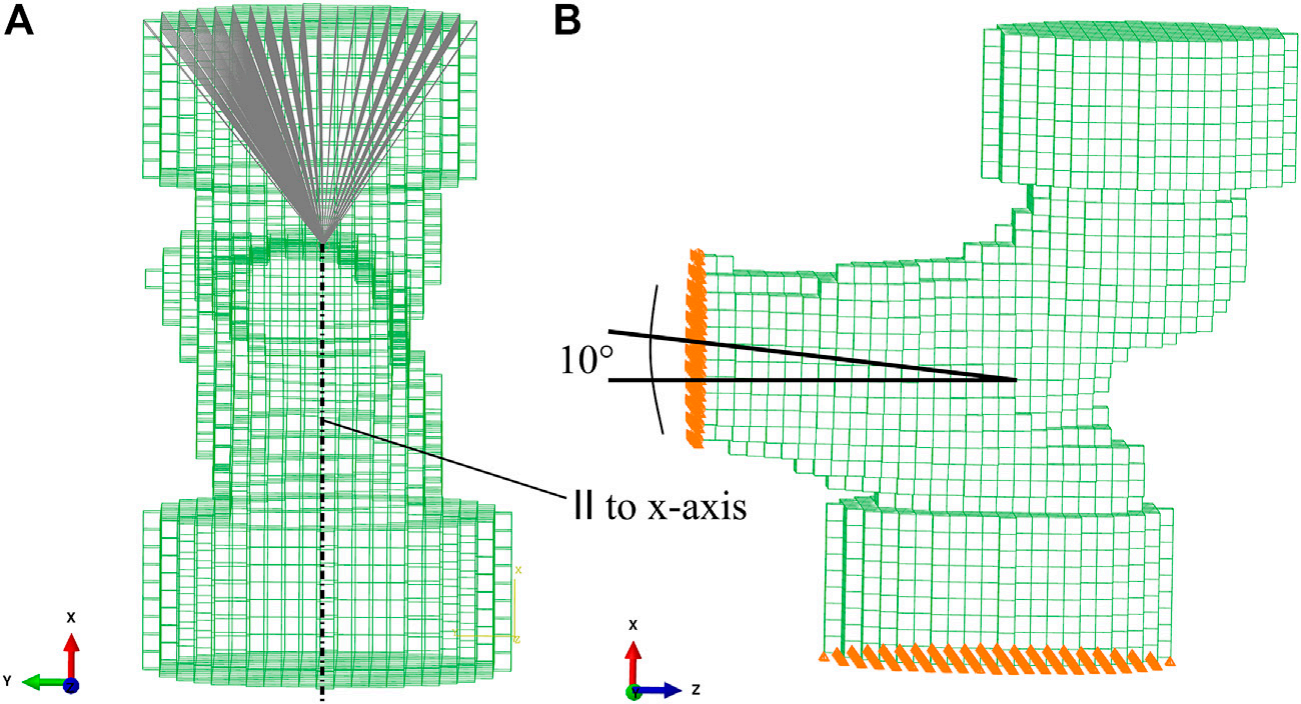
FE mesh with embedding caps. **(A)** Kinematic coupling between the driving node at the center of the femoral head and the upper surface of the embedding cap. **(B)** 10° inclination of the proximal shaft axis. Nodes on the distal cut surface were fully constrained. Nodes on the lower surface of the GT embedding were constrained in the vertical direction and free to move laterally.

The FE model was created based on the implicit coordinate system established above. The axes of the implicit coordinate system were used to transform the mask rigidly in a configuration mimicking a fall to the side with an impact on the greater trochanter (GT). In this configuration, the shaft is inclined by 10° with respect to the horizontal ground, as shown in Figure 4. The plane formed by shaft and neck axes is oriented vertically. This configuration corresponds to an internal rotation that compensates for anteversion and therefore models a side fall slightly oriented backward. Before converting the voxels in the mask to finite elements, the image was downsampled to a voxel size of 3 mm. This element size yielded a reasonable compromise between computation time and precision in the FE simulations (Dall’Ara et al., 2013; Panyasantisuk et al., 2018).

The material properties of bone were mainly governed by bone volume fraction (BV/TV). BV/TV was obtained by converting the local BMD values (Dall’Ara et al., 2013) (Supplementary Material: 1.2.4 BMD to BV/TV conversion). For each FE element, a spherical region of equivalent volume was defined in the original image. The BMD values were then averaged within the region and mapped to the transformed mask using the same rigid body transform as above.

An isotropic elastic-perfectly plastic material model was chosen, which includes an isotropic damage formulation with the cumulative plastic strain as the driving quantity for the damage variable (Schwiedrzik and Zysset, 2013). The isotropic damage variable can vary between 0 (undamaged) and 1 (fully damaged) and affects all components of the elastic stiffness tensor alike. Yielding was defined by the isotropic formulation of the generalized quadric criterion by Schwiedrzik et al. (2013). Elastic and yield constants were extrapolated from the experimental results by Rincón-Kohli and Zysset (2009) who tested human trabecular bone. A piecewise function as described by Dall’Ara et al. (2013) was used to scale elasticity and yield properties with BV/TV. This is a modification of the formulation for trabecular bone by Zysset (2003) and aims at getting more realistic properties for elements with BV/TV above 0.5, which contain a large fraction of cortical bone. The parameters of the piecewise function were chosen so as to obtain an elastic modulus of 24 GPa and tensile and compressive yield stresses of 199 MPa respectively 264 MPa for idealized pore-less bone (BV/TV = 1). These values may seem rather high for cortical bone tissue. However, given a typical BV/TV of 0.907 (Boughton et al., 2019), the elastic modulus reduces to 15.9 GPa and the tensile and compressive yield stresses to 131 MPa resp. 175 MPa, where the tensile and compressive yield strains remain constant at 0.83% respectively 1.1%. Overall, these numbers are in line with literature values (Reilly and Burstein, 1974; Cezayirlioglu et al., 1985; Bayraktar et al., 2004; Öhman et al., 2011). The material model is detailed in the Supplementary Material: 1.2.3 Constitutive model; Supplementary Figure S3.

To replicate the experimental setup by Dall’Ara et al. (2013), the most lateral aspect of the GT and the most medial part of the femoral head in the experimental position were embedded in polyurethane contained in a steel cylinder (Figure 4B). Since the embedding depth in the experiment was 10 mm, three to four layers of voxels were chosen as embedding depth in the FE model. The femur was cut one head radius distal to the LT. Voxels were directly converted into linear isotropic hexahedral (8-node linear brick—C3D8) finite elements. The FE models consisted in 8,500–15,500 elements and 10,500–18,800 nodes.

Nodes on the distal cut surface were fully constrained. Nodes on the lower surface of the GT embedding were constrained only in the vertical direction. A vertical displacement was assigned to a node in the head center. A kinematic coupling between the driving node in the head center and the upper surface of the embedding of the femoral head was introduced (Figure 4A), in order to simulate a possible sliding motion between the surface of the femoral head and the embedding polyurethane in the experiment (Panyasantisuk et al., 2018). The reaction force at the driving node was recorded for each increment in the FE analysis.

The primary outcome of the simulations was the strength of the proximal part of the femur with a dimension of force (N). With the perfectly plastic post-yield behavior, no clear maximum was reached in most force-displacement curves. The inclusion of softening in the post-yield domain, on the other hand, led to pronounced strain localization in several cases. Consequently, strength was defined as the reaction force on the driving node at the femoral head center when a displacement corresponding to 4% of the distance femoral head center-GT was reached. This definition corresponds to the one adopted by Orwoll et al. (2009) and Kopperdahl et al. (2014).

Simulations were run on Abaqus 2021.HF4 (Dassault Systèmes, Vélizy-Villacoublay, France) on a small local cluster. The cluster was comprised of Intel Xeon CPU E5-2690 v3 (2.60 GHz) processors, which ran Red Hat Enterprise Linux 8.4. Pre- and postprocessing operations were carried out on a single CPU, whereas Abaqus simulations were run on 8 CPUs. Total processing times varied between 3 and 9 min per model.

Likewise, FE models were created for a position mimicking the stance phase of the gait cycle. Details for this case and the corresponding results are included in the Supplementary Material: 1.2.2 Stance configuration; Supplementary Figures S2, S4, S5.

#### 2.9.3 Adjustment of bone mineral content

In the first iteration, QCT and 3D DXA data were processed independently through the FE pipeline using their original density values. At the same time, the voxel-based meshes were saved as image files with each voxel containing the local BMD value. The BMC was computed for each mesh as the volume integral of local BMD values over the whole volume of bone present in the respective mesh. As significant differences were discovered between the BMC values based on QCT and 3D DXA images in the foregoing analysis, two corrections were introduced for the BMC computed from the 3D DXA-based meshes: A “collective correction” to remove the calibration errors between the DXA and QCT machines and an “individual correction” to observe the effects of density distribution differences on strength outcomes based on QCT and 3D DXA respectively. The two following paragraphs explain the two corrections. Both corrections were computed for the sets of meshes representing fall and stance load cases separately. This was necessary, as the most distal part of the mesh differs between fall and stance, due to a different orientation of the femoral shaft with respect to the transverse plane along which the meshes were cut.

First, a linear regression analysis was performed between the BMC values computed from QCT and 3D DXA-based meshes. The linear regression equation was then used to estimate the BMC of the QCT-based mesh corresponding to the respective 3D DXA-based mesh ( $\hat{{BMC}_{QCT\;fall}}$ ). The ratio between estimated BMC of the QCT-based mesh and the BMC of the 3D DXA-based mesh ( $f_{collective}$ ) was then used to scale the local BMD values of all elements in the 3D DXA-based mesh. The equations below summarize the correction procedure.

$$\hat{{BMC}_{QCT\;fall}}=m∙{BMC}_{3D\;DXA\;fall}+b$$

$$f_{collective}=\frac{\hat{{BMC}_{QCT\;fall}}}{{BMC}_{3D\;DXA\;fall}}$$

$${BMD}_{3D\;DXA\;fall\;collective}^{i}=f_{collective}∙{BMD}_{3D\;DXA\;fall}^{i}for\;1\leq i\leq Nbr.\;bone\;elements$$

The FE simulation was rerun for the corrected 3D DXA-based meshes, and the corresponding femoral strength values were extracted as described above. Such a procedure, based on a calibration equation, could be applied in clinics to correct for the average density differences between QCT and 3D DXA images. In the following this set of simulations is called “collectively corrected simulations.”

For the second correction, the ratios of BMC computed from the QCT-based meshes to the BMC of their respective 3D DXA-based meshes were computed ( $f_{individual}$ ). The local BMD values of all elements in the 3D DXA-based mesh were scaled with the so-obtained constant scaling factor resulting in pairs of corresponding QCT- and 3D DXA-based meshes with the same BMC value. The correction of a given 3D DXA-based mesh with respect to its respective QCT-based mesh is summarized in the equations below.

$$f_{individual}=\frac{{BMC}_{QCT\;fall}}{{BMC}_{3D\;DXA\;fall}}$$

$${BMD}_{3D\;DXA\;fall\;individual}^{i}=f_{individual}∙{BMD}_{3D\;DXA\;fall}^{i}for\;1\leq i\leq Nbr.\;bone\;elements$$

The FE simulations were repeated with this set of corrected 3D DXA-based meshes and femoral strength was extracted as described above. In addition, the spatial distribution of damage was extracted at the point of the estimated femoral strength. This second correction aimed to isolate the effect of differences in shape and density distribution between QCT- and 3D DXA-based simulations on the strength and fracture patterns. This set of simulations is termed “individually corrected simulations.”

#### 2.9.4 Statistical analysis of FE-based strength outcomes

We performed linear regression analysis between QCT- and 3D DXA-based femoral strength measures for uncorrected 3D DXA-based simulations and the collectively and individually corrected sets of 3D DXA-based simulations. The coefficient of determination and the concordance correlation coefficient (Lin, 1989) were computed. A bootstrapping approach was applied to obtain confidence intervals (95%) for the coefficient of determination. The linear regression analysis was repeated for QCT-based strengths versus DXA-based aBMD. The coefficient of determination and its confidence interval were computed using the same approach as above. We determined the means and standard deviations of predicted and predictor variables and the standard error of the estimate (SEE) for the linear regressions. In addition, we calculated the mean absolute error (MAE) between paired 3D DXA and QCT-based strengths and the corresponding coefficients of variation (CV of MAE).

Statistical analysis was performed in Python 3.10. using the package SciPy (version 1.9.0).

### 2.10 Spatial distribution of damage

This analysis was performed based on the individually corrected simulations, where the BMC of 3D DXA-based meshes was adjusted to the BMC of QCT-based meshes for the simulation. The spatial distribution of damage (damage map) was extracted in an FE postprocessing step for both QCT and 3D DXA-based simulations to visualize the effect of shape and density distribution differences between QCT-based and 3D DXA-based simulations on the fracture pattern. We created damage maps for the point where strength was reached in the force-displacement curve of each case. Each damage map consisted of a 3D image of the corresponding femur at an isotropic resolution of 3 mm, where each voxel contained a value between 0 (undamaged) and 1 (fully damaged). All QCT damage maps were registered non-rigidly to the standard femur. Binary masks of both target and moving images were used to compute the registration to avoid the influence of damage values in the registration. After aligning the damage maps, the damage values for each voxel were averaged to produce a mean QCT damage map. The same was repeated for the 3D DXA damage maps.

## 3 Results

### 3.1 Example

**FIGURE 5:**
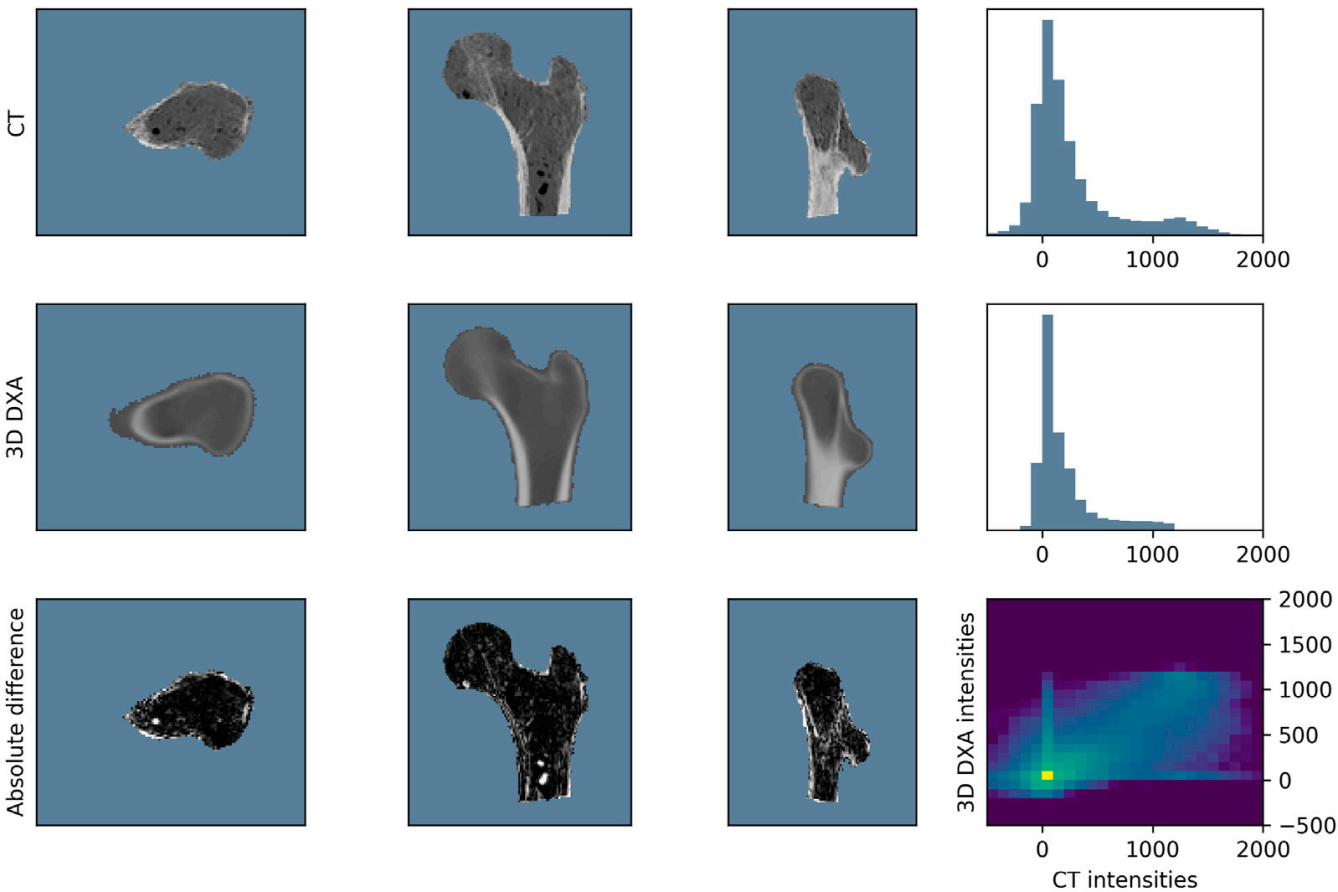
Example with average registration performance (MI = 0.10, *r* ^2^ = 0.52, dice = 0.91).

Figure 5 compares the BMD values in the CT scan (first row) with those in the 3D DXA reconstruction (second row). The third row shows the absolute BMD difference between the two images and the joint histogram, on which the MI score is based. The average dice score between segmentation pairs of the whole dataset is 0.89±0.02 .

### 3.2 Geometry

The volume of the proximal femur is generally smaller in the 3D DXA than in the CT image, with an average difference of 3% in the lesser trochanter, 11% in the greater trochanter, 9% in the neck, 7% in these three regions together and 13% in the head. All differences are significant with *p*-values < 0.01.

The results for the average and maximal surface distances are shown in Table 2 for the regions presented above and for the whole reconstructed proximal femur. For comparison, Table 2 also shows the results from Humbert et al. (2017), who used a comparable method. The average distances are lower than our results by 18%–21%, except for the femoral head, where they are larger by 42%. Furthermore, the maximum values Humbert et al. (2017) reported are higher for all regions. All differences are significant with *p*-values < 0.01.

**TABLE 2:** Mean and standard deviation of the average and maximal surface distances. LT and GT stand for lesser and greater trochanter, respectively, and were named shaft and trochanter by Humbert et al. (2017).

	Evaluation		Humbert et al. (2017)	
	Avg. dist. (mm)	Max. dist. (mm)	Avg. dist. (mm)	Max. dist. (mm)
Neck	1.06 ± 0.23	3.09 ± 0.76	0.87	8.31
LT/Shaft	1.22 ± 0.23	4.47 ± 1.26	1.00	6.55
GT/Trochanter	1.18 ± 0.25	4.23 ± 1.04	0.89	7.12
Total (3 ROIs)	1.17 ± 0.18	4.84 ± 1.21	0.93	8.31
Head	1.24 ± 0.49	3.81 ± 1.21	1.77	8.46
Total proximal	1.18 ± 0.21	5.00 ± 1.23	N/A	N/A

We observe that the average surface distance corresponds roughly to the image resolution (1 mm). To estimate the registration precision, we resampled one CT image with shifts of 1/3 and 2/3 voxel spacing along the different axes and computed the registration anew. The resulting differences in the average surface distance are smaller than 1/20 voxel spacing.

**FIGURE 6:**
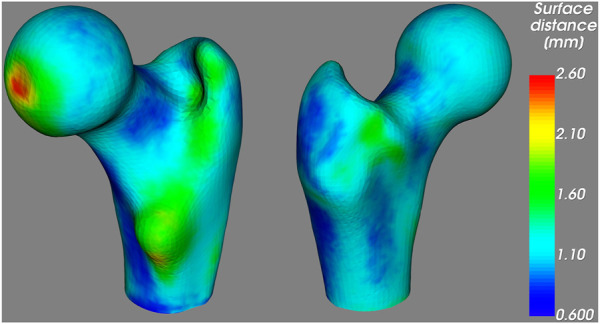
Average unsigned distance between the proximal femur surfaces obtained from the CT and the 3D DXA images, mapped on the standard femur.

The spatial distribution of the surface distances is illustrated in Figure 6, where we observe low distances around the femoral neck, coherently with the results above. The largest distances are found at the fovea for the ligament of the femoral head; however, this region is particularly prone to segmentation mistakes due to the junction between bone and ligament. Large distances are also found around the lesser trochanter, which could be responsible for the comparatively large average distance observed in the shaft region in Table 2.

### 3.3 Bone mineral density

In Table 3, the integral BMD results for the CT and 3D DXA images are presented, as well as the density differences and the correlation coefficients. Although all regions show a high correlation between CT and 3D DXA values, we observe large differences in the absolute values. Indeed, the average 3D DXA BMD values are systematically lower than the CT BMD values by up to 30%.

**TABLE 3:** Mean and standard deviation of the integral BMD results (mg/cm^3^). The density differences and correlation coefficients associated with *p*-values < 0.01 are marked with two stars. The remaining differences were associated with *p*-values > 0.05. LT and GT stand for lesser and greater trochanter, respectively, and were named shaft and trochanter by Humbert et al. (2017).

	BMD (mg/cm^3^)	CT	3D DXA	Difference	*r* ^2^**
Evaluation	Neck	252 ± 73	188 ± 65	−63 ± 28**	0.85
	LT/Shaft	312 ± 8	235 ± 73	−78 ± 27**	0.91
	GT/Trochanter	184 ± 64	128 ± 55	−56 ± 20**	0.92
	Total (3 ROIs)	250 ± 73	185 ± 62	−64 ± 21**	0.93
	Head	197 ± 71	162 ± 54	−35 ± 28**	0.87
Humbert et al. (2017)	Neck	300 ± 35	312 ± 35	13 ± 27	0.85
	LT/Shaft	339 ± 39	353 ± 42	14 ± 28	0.88
	GT/Trochanter	216 ± 27	220 ± 29	4 ± 20	0.86
	Total (3 ROIs)	284 ± 32	294 ± 34	10 ± 21	0.90

As a comparison, Table 3 also shows the results from Humbert et al. (2017) for the same regions except the femoral head. The correlation coefficients are very similar to the ones obtained in our evaluation. However, we observe a systematic density difference in the opposite direction, with larger densities in the 3D DXA than in the CT images. The DXA and the CT densities are significantly larger (*p* < 0.01) in the results from Humbert et al. (2017) compared to our study, with a particularly large difference between DXA densities.

Corresponding results for the integral BMC are shown in Table 4. As in the BMD results, the 3D DXA shows systematically lower values than the CT image in our evaluation, while the results from Humbert et al. (2017) present a systematic difference in the other direction.

**TABLE 4:** Mean and standard deviation of the integral BMC results (g). The content differences and correlation coefficients associated with *p*-values < 0.01 are marked with two stars. The remaining differences were associated with *p*-values > 0.05. LT and GT stand for lesser and greater trochanter, respectively, and were named shaft and trochanter by Humbert et al. (2017).

	BMC (g)	CT	3D DXA	Difference	*r* ^2^**
Evaluation	Neck	4.5 ± 1.8	3.1 ± 1.5	−1.4 ± 0.5**	0.95
	LT/Shaft	13.7 ± 5.2	10.0 ± 4.3	−3.7 ± 1.2**	0.97
	GT/Trochanter	7.7 ± 3.5	4.9 ± 2.7	−2.8 ± 1.0**	0.96
	Total (3 ROIs)	25.9 ± 10.3	18.0 ± 8.4	−7.9 ± 2.5**	0.97
	Head	8.6 ± 4.0	6.4 ± 3.1	−2.3 ± 1.3**	0.92
Humbert et al. (2017)	Neck	4.4 ± 0.8	4.5 ± 0.8	0.1 ± 0.4	0.94
	LT/Shaft	12.9 ± 2.2	13.1 ± 2.4	0.2 ± 1.0	0.96
	GT/Trochanter	7.4 ± 1.5	7.3 ± 1.5	−0.1 ± 0.7	0.94
	Total (3 ROIs)	24.7 ± 4.4	25.0 ± 4.6	0.2 ± 1.7	0.96

### 3.4 BMD distribution

Although the overall density results shown in Table 4 correlated quite well, we see in Table 5 that the BMD values of the individual voxels show much lower correlation coefficients, especially in the trochanter and head regions. Thus, the density differences do not seem to be homogeneously distributed. The correlation is significant with *p* < 0.01 for all pairs, except for the correlation in the head region of one femur. Indeed, the CT scan of this femur shows a region of high densities inside the femoral head which is not present in the 3D DXA image.

**TABLE 5:** Mean and standard deviation of the voxel BMD correlation coefficient.

	Voxel-voxel BMD correlation (*r* ^2^)
Neck	0.47 ± 0.10
Lesser trochanter	0.51 ± 0.10
Greater trochanter	0.27 ± 0.12
Total (3 ROIs)	0.48 ± 0.09
Head	0.21 ± 0.11

**FIGURE 7:**
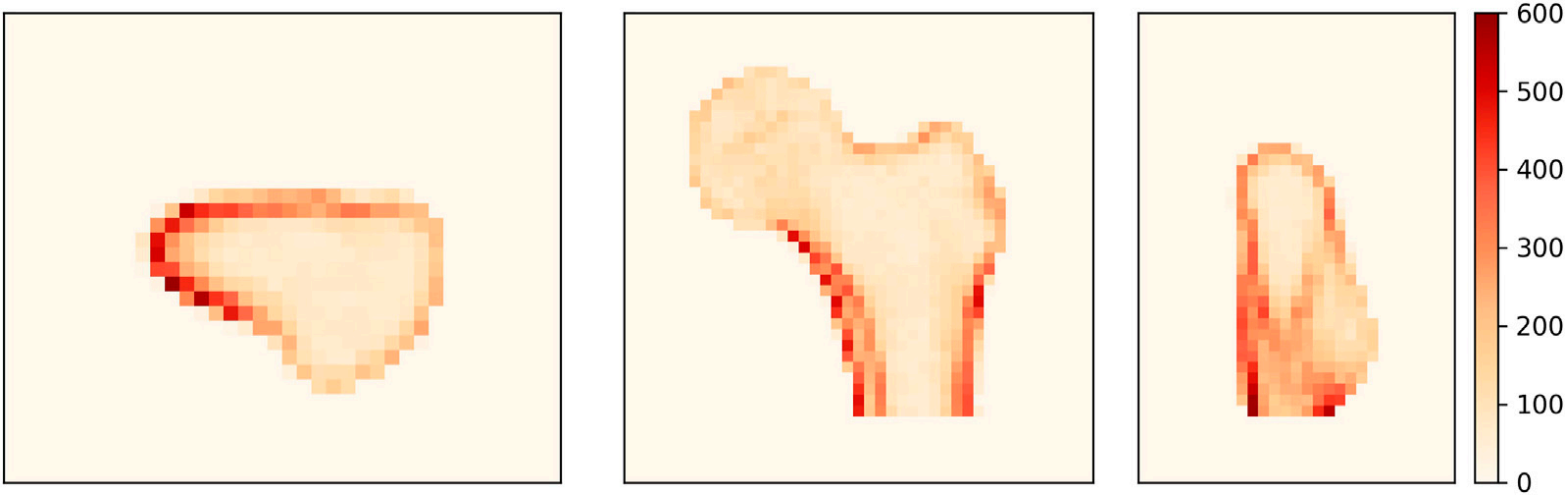
Average unsigned BMD difference between CT and 3D DXA (mg/cm^3^) shown on an axial (left), a coronal (center) and a sagittal (right) plane of the standard femur.

To identify the regions where the density distribution is not properly reconstructed, we analyze the distribution of the density differences between the CT image and the 3D DXA reconstruction. Figure 7 shows the unsigned BMD difference for three image slices. The largest differences are found in the shaft cortex and the lower neck cortex, with differences of up to 620 mg/cm^3^. The two lines visible in the shaft cortex could indicate that the cortical thickness or position does not match between CT and 3D DXA.

### 3.5 FE analysis

#### 3.5.1 Regression results and error computation

**FIGURE 8:**
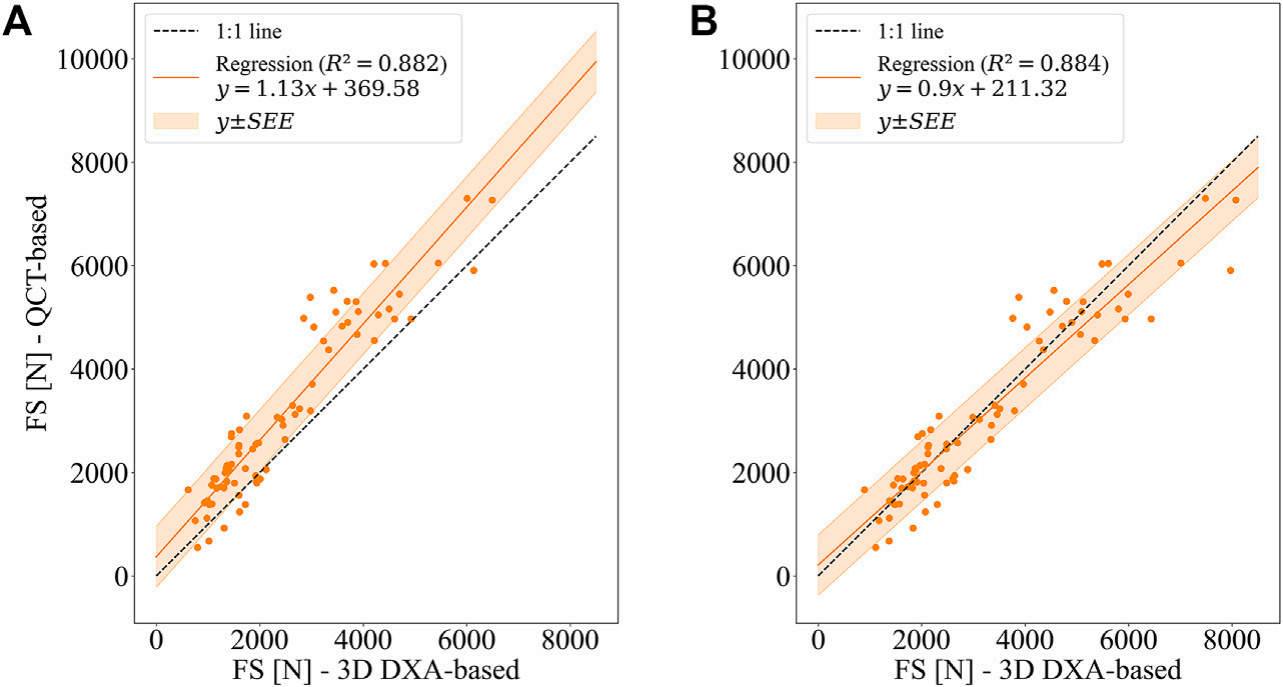
Linear regression between QCT-based and 3D DXA-based femoral strengths (FS) for the side fall configuration. **(A)** Uncorrected 3D DXA-based FE simulations **(B)** BMC of 3D DXA-based meshes adjusted to BMC of QCT-based meshes (collectively corrected 3D DXA-based FE simulations).

Mean femoral strengths computed based on QCT and 3D DXA were: 3,144 ± 1,686 N (QCT), 2,463 ± 1,406 N (uncorrected 3D DXA), 3,245 ± 1,754 N (collectively corrected 3D DXA), and 3,262 ± 1,793 N (individually corrected 3D DXA). Figure 8 shows the linear regression results for QCT and 3D DXA-based simulations. The uncorrected 3D DXA-based strength outcomes correlate well with the QCT-based strength [*r* ^2^ = 0.882, 95% CI (0.830, 0.918)]. A similar coefficient of determination (*r* ^2^ = 0.884) is obtained using the collectively corrected 3D DXA-based meshes, which account for average differences between the BMC values of QCT and 3D DXA. However, both these values are not significantly different from the coefficient of determination between DXA-based femoral neck (FN) aBMD and QCT-based strength [*r* ^2^ = 0.868, 95% CI (0.797, 0.913)].

The slope (1.13 ± 0.09) and the intercept (369.58 ± 269.2 N) of the linear relationship illustrate that the uncorrected 3D DXA-based simulations underestimate the strengths computed with the QCT images. It can also be noted that the slope is significantly different from unity, and the intercept is significantly larger than zero at a 95% confidence interval. This difference is not surprising given the overall shift in BMC values found in the preceding analysis.

The shift of the strength values is largely corrected when adjusting the BMC of 3D DXA-based meshes, either using the collective or the individual correction (Figure 8; Table 6). However, with both adjustments, the slope is still significantly different from 1, whereas the intercept is not significantly different from 0 at a 95% confidence interval. Table 6 summarizes the linear regression results, error measures, and concordance between 3D DXA and QCT-based FE analysis. As could be expected, the adjustments of BMC values for the 3D DXA-based meshes lead to an increase in the concordance coefficient from 0.880 (uncorrected) to 0.937 (collectively corrected), respectively 0.955 (individually corrected). Accordingly, the coefficient of variation of the mean absolute error (CV of MAE) decreases from 23.6% to 14.7%, respectively 11.7%, and the CV of SEE decreases from 18.7% to 18.5%, respectively 14.8%. Compared to the CV of the SEE of the relation between FN aBMD and QCT-based strength (19.8%), this indicates a possible advantage of 3D DXA-based strength over a simple regression between FN aBMD and QCT-based strength.

**TABLE 6:** Summary of linear regression results, error measures, and concordance.

	QCT vs. uncorrected 3D DXA-based simulations	QCT vs. collectively corrected 3D DXA-based simulations	QCT vs. individually corrected 3D DXA-based simulations	QCT-based simulation vs. DXA-based FN aBMD
N	77			
r^2^	0.882	0.884	0.926	0.868
Slope (95% CI)	1.13 ± 0.09 (-)	0.90 ± 0.08 (-)	0.90 ± 0.06 (-)	9,151 ± 822 [ $\frac{N}{\frac{g}{{cm}^{2}}}$ ]
Intercept (95% CI) (N)	369.6 ± 269.2	192.3 ± 219.5	211.3 ± 277.9	−3,102 ± 578
SEE (N)	588	582	466	621
Mean predictor strength (3D DXA) (N)	2,463 ± 1,406	3,245 ± 1754	3,262 ± 1793	N/A
Mean predicted strength (QCT) (N)	3,144 ± 1,686			
CV of SEE (%)	18.7	18.5	14.8	19.8
MAE (N)	742	461	369	N/A
CV of MAE (%)	23.6	14.7	11.7	N/A
Concordance correlation coefficient	0.880	0.937	0.955	N/A

It can be noted in Figure 8 that the scatter is substantially larger for high-strength femurs (>5,000 N) than for low-strength femurs. These cases contribute substantially to the MAE and CV. However, for identifying people at risk of fracture, performance in the low-strength range is more relevant than in the high-strength range.

The trends are essentially the same for the simulation results in the stance configuration. However, it can be highlighted that differences in the coefficient of determination (*r* ^2^) between 3D DXA-based regressions and FN aBMD-based regressions are more pronounced in the stance than in the side fall configuration. Indeed, the coefficient of determination is *r* ^2^ = 0.878 [95% CI (0.823, 0.920)] for the uncorrected 3D DXA-based meshes and *r* ^2^ = 0.831 [95% CI (0.753, 0.885)] for the regression between FN aBMD and QCT-based strength. Detailed results of the simulations in stance configuration can be found in Supplementary Table S2 and Supplementary Figure S4.

#### 3.5.2 Damage maps

**FIGURE 9:**
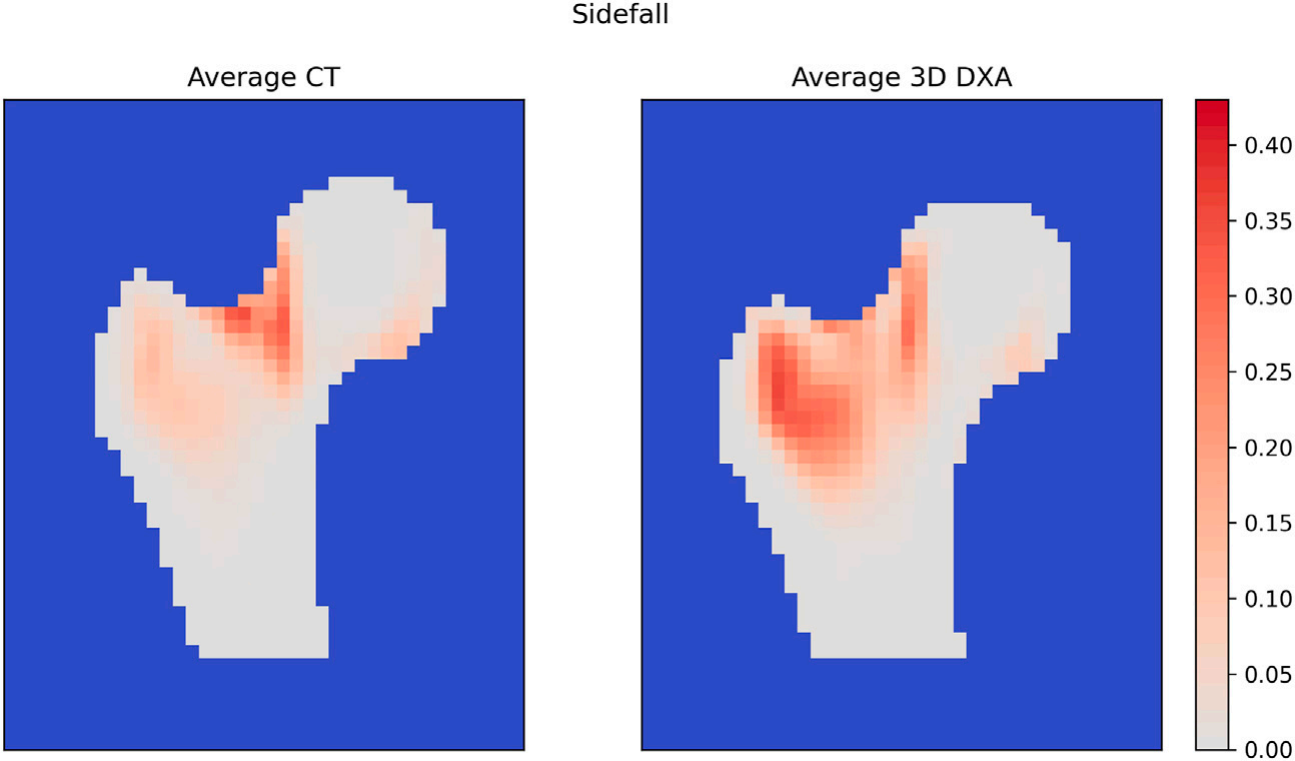
Average damage maps for QCT and 3D DXA mapped on the standard femur.

The average QCT and 3D DXA damage maps are shown in Figure 9 for the side fall configuration. In the QCT map, high damage values (>0.2) are concentrated in the upper neck region, while the 3D DXA damage map shows high values from the greater trochanter region up to the boundary between the neck and head. This broader spatial distribution of the 3D DXA damage could be due to the smoother density distribution in 3D DXA images. In contrast, the QCT densities show sharper density gradients between the head and neck, for example.

For the stance configuration (Supplementary Figure S5), the average QCT and 3D DXA damage maps were similar, with high damage values located mainly in the inferior part of the femoral neck.

## 4 Discussion

The 3D-Shaper software was able to reconstruct the geometry of the proximal femur from 2D DXA scans with average surface distances smaller than 2 mm for all femora but a slightly underestimated volume. Our results for the average surface distance were 18%–21% higher than those by Humbert et al. (2017), except in the femoral head, where they were 42% lower. The latter can be explained by the fact that the acetabulum covers the femoral head in the clinical images used by Humbert et al. (2017), while in our scans, the head is fully visible and can thus be reconstructed more accurately. Therefore, our results are likely to underestimate the average surface distance of the femoral head reconstruction from clinical DXA images. In all regions, the maximal distances were substantially smaller in our study. However, it should be noted that the maximal distance can be determined by a single outlier and is thus strongly dependent on the smoothness of the segmentation method, which was different in both evaluations.

While the density difference between QCT and 3D DXA BMC was small in Humbert et al. (2017), it was significant in our study. Part of this larger density gap can be attributed to the fact that Humbert et al. (2017) performed their evaluation on clinical images while we used *ex vivo* scans. The impact of this difference on QCT scans is limited, but DXA results depend on the soft tissue composition. Like previous *ex vivo* DXA studies, we scanned the femora between water bags to account for soft tissues. However, water is an adequate surrogate for lean but not for fat tissues, which have similar coefficients of attenuation at high but not at low energy (Jonson, 1993). The respective amount of each tissue type differs in every patient and is generally unknown, which is a well-known limitation of DXA scanners (Bolotin, 1998; Hakulinen et al., 2003) and makes any choice of soft tissue surrogate inexact. Since the attenuation coefficient of water differs slightly from the value used by the DXA software for soft tissues, the computed bone density is not entirely independent of water thickness. A small test with water bags of different thicknesses was conducted, which showed a few percent variations in the aBMD values. Nevertheless, using the same water bags for all DXA scans allowed to compare bone densities between femora without the uncertainty induced by variable soft tissue compositions and thicknesses, which is an advantage of *ex vivo* scans. Moreover, Humbert et al. (2017) used a liquid phantom (with K_2_HPO_4_) as calibration reference for their QCT device, while we used a solid phantom (with Ca_10_(PO_4_)_6_(OH)_2_). Since differences between the calibration results of liquid and solid phantoms have been reported (Faulkner et al., 1993), this might increase the differences between both studies. Despite the density gap, which shows that the calibration of QCT and DXA devices should be treated very carefully, we obtained a good correlation between QCT and 3D DXA BMC values.

The reconstruction of the density distribution seemed to be more problematic. As shown in the histogram comparison of a representative femur, the 3D DXA densities were smoother and missed the second peak, which typically corresponds to the cortical densities. Furthermore, the spatial density distribution analysis revealed a heterogeneous distribution of the density reconstruction error. High-density regions, such as the cortex, showed more variation than low-density regions. In particular we observed large density differences of up to 620 mg/cm^3^ in the neck cortex, which is a critical area for fractures. In addition, the low voxel BMD correlation values showed that the algorithm failed to reconstruct the inner structure of the bone at the level of detail of a CT scan. It should be noted that the density reconstruction in the femoral head would be even more challenging for clinical DXA scans. Indeed, the acetabulum would cover the femoral head, which was not the case in our *ex vivo* scans. This part of the reconstruction could be improved by mapping the density distribution from a standard or similar CT onto the 3D DXA image.

Bone volume fraction, which is closely related to vBMD in clinical CT images, dictates the mechanical properties of bone to a high degree (Maquer et al., 2015; Musy et al., 2017). Consequently, it is not surprising that a shift in BMC led to a shift in bone strength estimated by FE analysis, as seen in Figure 8. The largest absolute errors were produced in the strength range above 4,000 N, which is the uncritical range regarding fracture risk (Kopperdahl et al., 2014). However, at first sight the relative errors of 3D DXA-based strengths with respect to QCT-based strengths seem to be elevated in the low strength region, which may be a limiting factor for the reliability of the 3D DXA-based strength analysis for fracture risk prediction. The match between strength values obtained from QCT and 3D DXA was improved by adjusting the BMC of the 3D DXA-based meshes to the one based on QCT. However, neither the regression between the uncorrected 3D DXA-based simulations and the QCT-based simulations nor the one between the collectively corrected 3D DXA-based and the QCT-based simulations were significantly better than the regression between FN aBMD and strengths obtained from QCT-based simulations. Nevertheless, the 3D DXA allows calculating a femoral strength from a DXA scan that can be compared directly to standardized QCT-based definitions of fragile, low, or normal strength and could be used directly within a more “mechanistic” model for fracture risk estimation.

Furthermore, the differences in BMD distribution led to different fracture patterns in 3D DXA-based and QCT-based simulations in the side fall configuration. Fractures were predicted in the pertrochanteric region by 3D DXA-based simulations and in the femoral neck region by QCT-based simulations. These different fracture patterns can be explained by the density distribution differences between 3D DXA and QCT. Indeed, as mentioned before, the density distribution is smoother in the 3D DXA and does not show the same level of detail as the QCT scan.

Our evaluation is limited by the following aspects. Due to the nature of the dataset, the age range of the subjects covers mainly the elderly population, with only a few middle-aged donors. Thus, these results should be verified on younger subjects. As mentioned above, scanning the bones between bags of water produces different results than clinical DXA scans, while reducing the uncertainty. In addition, the incomplete coverage of the CT scans by the calibration phantom forced us to extrapolate the phantom HU values for 11 images. However, excluding these images from the dataset did not change the results significantly. A further limitation is the presence of air bubbles in the proximal part of the femur, although mostly located in the medullary canal and therefore not included in the analysis.

In general, the reconstruction algorithm depends on the quality of the dataset used to create the statistical model of shape and density. In the case of 3D-Shaper, Humbert et al. (2017) used 111 subjects scanned in two centers. It is therefore likely that diversifying and increasing the number of bone samples in that dataset would improve the generalization ability of the algorithm. In addition, other reconstruction algorithms should be considered to improve the spatial distribution of the densities, such as the method by (Ahmad et al., 2010), which uses four DXA images taken at different angles to reconstruct a 3D image of the femur. The repeatability has not been addressed in this work, but was investigated by a previous study (O’Rourke et al., 2021) and will be the object of a future analysis of repeated DXA scans in our laboratory.

In conclusion, the algorithm implemented in 3D-Shaper performs well in reconstructing the geometry of the femur but requires careful calibration of BMD and shows limitations in recovering the spatial BMD distributions. Therefore, the potential of 3D-Shaper for assessing changes in spatial BMD distribution, for example, with age or with medication, may be limited. Despite distinct damage distributions in the FE analyses, the 3D DXA-based FE strength outcome correlated well with the QCT-based strengths, although the improvement with respect to aBMD was not significant. With the clinical application in mind, future work needs to address the repeatability of the 3D DXA technique and its potential to outperform simple DXA outcomes to predict, fracture risk.

## Data Availability

The raw data supporting the conclusion of this article will be made available by the authors, without undue reservation.

## References

[B1] Ahmad O. Ramamurthi K. Wilson K. E. Engelke K. Prince R. L. Taylor R. H. (2010). Volumetric DXA (VXA): A new method to extract 3D information from multiple *in vivo* DXA images. J. Bone Min. Res. 25, 2744–2751. 10.1002/jbmr.140 20533301

[B2] Bayraktar H. H. Morgan E. F. Niebur G. L. Morris G. E. Wong E. K. Keaveny T. M. (2004). Comparison of the elastic and yield properties of human femoral trabecular and cortical bone tissue. J. Biomech. 37, 27–35. 10.1016/S0021-9290(03)00257-4 14672565

[B3] Bhattacharya P. Altai Z. Qasim M. Viceconti M. (2019). A multiscale model to predict current absolute risk of femoral fracture in a postmenopausal population. Biomech. Model Mechanobiol. 18, 301–318. 10.1007/s10237-018-1081-0 30276488 PMC6418062

[B4] Boehm H. F. Horng A. Notohamiprodjo M. Eckstein F. Burklein D. Panteleon A. (2008). Prediction of the fracture load of whole proximal femur specimens by topological analysis of the mineral distribution in DXA-scan images. Bone 43, 826–831. 10.1016/j.bone.2008.07.244 18723137

[B5] Bolotin H. H. (1998). A new perspective on the causal influence of soft tissue composition on DXA-measured *in vivo* bone mineral density. J. Bone Min. Res. 13, 1739–1746. 10.1359/jbmr.1998.13.11.1739 9797483

[B6] Boughton O. R. Ma S. Cai X. Yan L. Peralta L. Laugier P. (2019). Computed tomography porosity and spherical indentation for determining cortical bone millimetre-scale mechanical properties. Sci. Rep. 9, 7416. 10.1038/s41598-019-43686-6 31092837 PMC6520408

[B7] Bouxsein M. L. Zysset P. Glüer C. C. McClung M. Biver E. Pierroz D. D. (2020). Perspectives on the non-invasive evaluation of femoral strength in the assessment of hip fracture risk. Osteoporos. Int. 31, 393–408. 10.1007/s00198-019-05195-0 31900541

[B8] Cezayirlioglu H. Bahniuk E. Davy D. T. Heiple K. G. (1985). Anisotropic yield behavior of bone under combined axial force and torque. J. Biomech. 18, 61–69. 10.1016/0021-9290(85)90045-4 3980489

[B9] Chandran V. Reyes M. Zysset P. (2017). A novel registration-based methodology for prediction of trabecular bone fabric from clinical QCT: A comprehensive analysis. PLoS ONE 12, e0187874. 10.1371/journal.pone.0187874 29176881 PMC5703488

[B10] Dall’Ara E. Luisier B. Schmidt R. Kainberger F. Zysset P. Pahr D. (2013). A nonlinear QCT-based finite element model validation study for the human femur tested in two configurations *in vitro* . Bone 52, 27–38. 10.1016/j.bone.2012.09.006 22985891

[B11] Ehlke M. Ramm H. Lamecker H. Hege H.-C. Zachow S. (2013). Fast generation of virtual X-ray images for reconstruction of 3D anatomy. IEEE Trans. Vis. Comput. Graph. 19, 2673–2682. 10.1109/TVCG.2013.159 24051834

[B12] Engelke K. Lang T. Khosla S. Qin L. Zysset P. Leslie W. D. (2015). Clinical use of quantitative computed tomography (QCT) of the hip in the management of osteoporosis in adults: the 2015 ISCD official positions—Part I. J. Clin. Densitom. 18, 338–358. 10.1016/j.jocd.2015.06.012 26277851

[B13] Faulkner K. G. Glüer C. C. Grampp S. Genant H. K. (1993). Cross-calibration of liquid and solid QCT calibration standards: Corrections to the UCSF normative data. Osteoporos. Int. 3, 36–42. 10.1007/BF01623175 8422515

[B14] Grassi L. Väänänen S. P. Ristinmaa M. Jurvelin J. S. Isaksson H. (2017). Prediction of femoral strength using 3D finite element models reconstructed from DXA images: validation against experiments. Biomech. Model Mechanobiol. 16, 989–1000. 10.1007/s10237-016-0866-2 28004226 PMC5422489

[B15] Grassi L. Fleps I. Sahlstedt H. Väänänen S. P. Ferguson S. J. Isaksson H. (2021). Validation of 3D finite element models from simulated DXA images for biofidelic simulations of sideways fall impact to the hip. Bone 142, 115678. 10.1016/j.bone.2020.115678 33022451

[B16] Hakulinen M. A. Saarakkala S. T yr s J. Kr ger H. Jurvelin J. S. (2003). Dual energy x-ray laser measurement of calcaneal bone mineral density. Phys. Med. Biol. 48, 1741–1752. 10.1088/0031-9155/48/12/305 12870580

[B17] Holzer G. von Skrbensky G. Holzer L. A. Pichl W. (2009). Hip fractures and the contribution of cortical versus trabecular bone to femoral neck strength. J. Bone Mineral Res. 24, 468–474. 10.1359/jbmr.081108 19016592

[B18] Humbert L. Martelli Y. Fonolla R. Steghofer M. Di Gregorio S. Malouf J. (2017). 3D-DXA: Assessing the femoral shape, the trabecular macrostructure and the cortex in 3D from DXA images. IEEE Trans. Med. Imaging 36, 27–39. 10.1109/TMI.2016.2593346 27448343

[B19] Jonson R. (1993). Mass attenuation coefficients, quantities and units for use in bone mineral determinations. Osteoporos. Int. 3, 103–106. 10.1007/BF01623381 8453189

[B20] Kanis J. A. Norton N. Harvey N. C. Jacobson T. Johansson H. Lorentzon M. (2021). Scope 2021: A new scorecard for osteoporosis in europe. Arch. Osteoporos. 16, 82. 10.1007/s11657-020-00871-9 34080059 PMC8172408

[B21] Khoo B. C. C. Brown K. Cann C. Zhu K. Henzell S. Low V. (2009). Comparison of QCT-derived and DXA-derived areal bone mineral density and T scores. Osteoporos. Int. 20, 1539–1545. 10.1007/s00198-008-0820-y 19107384

[B22] Klein S. Staring M. Murphy K. Viergever M. A. Pluim J. (2010). Elastix: A toolbox for intensity-based medical image registration. IEEE Trans. Med. Imaging 29, 196–205. 10.1109/TMI.2009.2035616 19923044

[B23] Kopperdahl D. L. Aspelund T. Hoffmann P. F. Sigurdsson S. Siggeirsdottir K. Harris T. B. (2014). Assessment of incident spine and hip fractures in women and men using finite element analysis of CT scans: Incident fracture assessment using fea of CT scans. J. Bone Min. Res. 29, 570–580. 10.1002/jbmr.2069 PMC3925753 23956027

[B24] Langton C. M. Pisharody S. Keyak J. H. (2009). Generation of a 3D proximal femur shape from a single projection 2D radiographic image. Osteoporos. Int. 20, 455–461. 10.1007/s00198-008-0665-4 18563512

[B25] Lewiecki M. Betah D. Humbert L. Libanati C. Oates M. Shi Y. (2022). “Comparison of romosozumab and teriparatide effects on cortical and trabecular bone using 3D modeling from DXA images in postmenopausal women transitioning from bisphosphonate therapy,” in Presented at the 2022 Annual Meeting of the American Society for Bone and Mineral Research (Austin, Texas: Wiley).

[B26] Lin L. I.-K. (1989). A concordance correlation coefficient to evaluate reproducibility. Biometrics 45, 255. 10.2307/2532051 2720055

[B27] Maquer G. Musy S. N. Wandel J. Gross T. Zysset P. K. (2015). Bone volume fraction and fabric anisotropy are better determinants of trabecular bone stiffness than other morphological variables: The best determinants of trabecular bone stiffness. J. Bone Min. Res. 30, 1000–1008. 10.1002/jbmr.2437 25529534

[B28] McCloskey E. Johansson H. Oden A. Kanis J. A. (2012). Fracture risk assessment. Clin. Biochem. 45, 887–893. 10.1016/j.clinbiochem.2012.05.001 22579965

[B29] Musy S. N. Maquer G. Panyasantisuk J. Wandel J. Zysset P. K. (2017). Not only stiffness, but also yield strength of the trabecular structure determined by non-linear µFE is best predicted by bone volume fraction and fabric tensor. J. Mech. Behav. Biomed. Mater. 65, 808–813. 10.1016/j.jmbbm.2016.10.004 27788473

[B30] O’Rourke D. Beck B. R. Harding A. T. Watson S. L. Pivonka P. Martelli S. (2021). Assessment of femoral neck strength and bone mineral density changes following exercise using 3D-DXA images. J. Biomech. 119, 110315. 10.1016/j.jbiomech.2021.110315 33636460

[B31] O’Rourke D. Beck B. R. Harding A. T. Watson S. L. Pivonka P. Martelli S. (2022). Geometry and bone mineral density determinants of femoral neck strength changes following exercise. Biomech. Model Mechanobiol., 1–10. 10.1007/s10237-022-01642-w PMC9958140 36271264

[B32] Öhman C. Baleani M. Pani C. Taddei F. Alberghini M. Viceconti M. (2011). Compressive behaviour of child and adult cortical bone. Bone 49, 769–776. 10.1016/j.bone.2011.06.035 21763479

[B33] Orwoll E. S. Marshall L. M. Nielson C. M. Cummings S. R. Lapidus J. Cauley J. A. (2009). For the osteoporotic fractures in men (MrOS) study GroupFinite element analysis of the proximal femur and hip fracture risk in older men. J. Bone Mineral Res. 24, 475–483. 10.1359/jbmr.081201 PMC2659519 19049327

[B34] Panyasantisuk J. Dall’Ara E. Pretterklieber M. Pahr D. H. Zysset P. K. (2018). Mapping anisotropy improves QCT-based finite element estimation of hip strength in pooled stance and side-fall load configurations. Med. Eng. Phys. 59, 36–42. 10.1016/j.medengphy.2018.06.004 30131112

[B35] Reilly D. T. Burstein A. H. (1974). The elastic and ultimate properties of compact bone tissue. J. Biomech. 8, 393–405. 10.1016/0021-9290(75)90075-5 1206042

[B36] Reyneke C. J. F. Luthi M. Burdin V. Douglas T. S. Vetter T. Mutsvangwa T. E. M. (2019). Review of 2-D/3-D reconstruction using statistical shape and intensity models and X-ray image synthesis: Toward a unified framework. IEEE Rev. Biomed. Eng. 12, 269–286. 10.1109/RBME.2018.2876450 30334808

[B37] Rincón-Kohli L. Zysset P. K. (2009). Multi-axial mechanical properties of human trabecular bone. Biomech. Model Mechanobiol. 8, 195–208. 10.1007/s10237-008-0128-z 18695984

[B38] Roberts B. J. Thrall E. Muller J. A. Bouxsein M. L. (2010). Comparison of hip fracture risk prediction by femoral aBMD to experimentally measured factor of risk. Bone 46, 742–746. 10.1016/j.bone.2009.10.020 19854307

[B39] Ronneberger O. Fischer P. Brox T. (2015)U-Net: Convolutional Networks for Biomedical Image Segmentation,” in Editors Navab N. Hornegger J. Wells W. Frangi A. Medical Image Computing and Computer-Assisted Intervention – MICCAI 2015(London, United Kingdom: Lecture Notes in Computer Science, Springer, Cham) 9351. 10.1007/978-3-319-24574-4_28

[B40] Ruiz Wills C. Olivares A. L. Tassani S. Ceresa M. Zimmer V. González Ballester M. A. (2019). 3D patient-specific finite element models of the proximal femur based on DXA towards the classification of fracture and non-fracture cases. Bone 121, 89–99. 10.1016/j.bone.2019.01.001 30611923

[B41] Schechner Z. Luo G. Kaufman J. J. Siffert R. S. (2010). A Poisson process model for hip fracture risk. Med. Biol. Eng. Comput. 48, 799–810. 10.1007/s11517-010-0638-6 20524073

[B42] Schwiedrzik J. J. Zysset P. K. (2013). An anisotropic elastic-viscoplastic damage model for bone tissue. Biomech. Model Mechanobiol. 12, 201–213. 10.1007/s10237-012-0392-9 22527365

[B43] Schwiedrzik J. J. Wolfram U. Zysset P. K. (2013). A generalized anisotropic quadric yield criterion and its application to bone tissue at multiple length scales. Biomech. Model Mechanobiol. 12, 1155–1168. 10.1007/s10237-013-0472-5 23412886

[B44] Thevenot J. Koivumäki J. Kuhn V. Eckstein F. Jämsä T. (2014). A novel methodology for generating 3D finite element models of the hip from 2D radiographs. J. Biomech. 47, 438–444. 10.1016/j.jbiomech.2013.11.004 24290135

[B45] Unser M. Thevenaz P. (2000). Optimization of mutual information for multiresolution image registration. IEEE Trans. Image Process. 9, 2083–2099. 10.1109/83.887976 18262946

[B46] Unser M. (1999). Splines: A perfect fit for signal and image processing. IEEE Signal Process. Mag. 16, 22–38. 10.1109/79.799930

[B47] Väänänen S. P. Grassi L. Flivik G. Jurvelin J. S. Isaksson H. (2015). Generation of 3D shape, density, cortical thickness and finite element mesh of proximal femur from a DXA image. Med. Image Anal. 24, 125–134. 10.1016/j.media.2015.06.001 26148575

[B48] Whitmarsh T. Humbert L. De Craene M. Del Rio Barquero L. M. Frangi A. F. (2011). Reconstructing the 3D shape and bone mineral density distribution of the proximal femur from dual-energy X-ray absorptiometry. IEEE Trans. Med. Imaging 30, 2101–2114. 10.1109/TMI.2011.2163074 21803681

[B49] Winzenrieth R. Kostenuik P. Boxberger J. Wang Y. Humbert L. (2022). Proximal femur responses to sequential therapy with abaloparatide followed by alendronate in postmenopausal women with osteoporosis by 3D modeling of hip dual‐energy X‐ray absorptiometry (DXA). JBMR Plus 6, e10612. 10.1002/jbm4.10612 35434451 PMC9009108

[B50] Zebaze R. Seeman E. (2015). Cortical bone: A challenging geography: A challenging geography. J. Bone Min. Res. 30, 24–29. 10.1002/jbmr.2419 25431247

[B51] Zheng G. Gollmer S. Schumann S. Dong X. Feilkas T. González Ballester M. A. (2009). A 2D/3D correspondence building method for reconstruction of a patient-specific 3D bone surface model using point distribution models and calibrated X-ray images. Med. Image Anal. 13, 883–899. 10.1016/j.media.2008.12.003 19162529

[B52] Zysset P. K. (2003). A review of morphology–elasticity relationships in human trabecular bone: theories and experiments. J. Biomech. 36, 1469–1485. 10.1016/S0021-9290(03)00128-3 14499296

