# Supplementary material for: 2D-3D reconstruction of the proximal femur from DXA scans: Evaluation of the 3D-Shaper software
Source: Front Bioeng Biotechnol. 2023 Mar 1;11:1111020. doi: 10.3389/fbioe.2023.1111020 (PMC10014626; doi:10.3389/fbioe.2023.1111020)
Supplement: Supplementary file 1 [file DataSheet1.pdf]

Supplementary Material

1 Materials and Methods

1.1 Neural network for QCT segmentation

The neural network used for the QCT segmentation is a 3D version of the well-known U-Net architecture (Ronneberger et al., 2015). With respect to the original model, the number of convolutional layers was reduced from 23 to 14 to decrease the number of trainable parameters. The detailed architecture is illustrated in the Supplementary Figure S1. For the convolutional layers, the original kernel size (3x3) was extended to 3D (3x3x3). The convolution layers were padded to preserve the image size and followed by a rectified linear unit (ReLU). The max pooling layers had a kernel size 2x2x2 and stride 2 to halve the image size in all three dimensions. A dropout layer with rate 0.5 was introduced in the middle of the network to prevent overfitting. The upsampling layers had a size 2x2x2 to double the image size in all three dimensions. The output layer consisted of a final convolutional layer with kernel size 1x1x1, followed by a sigmoid activation function.
The model was trained with image-segmentation pairs for 80 epochs, using the dice coefficient as loss function and the Adam algorithm as optimizer (Kingma and Ba, 2017).

1.2 FE model

1.2.1 Implicit coordinate system of the proximal part of the femur

In order to transform the mask of the femur to the orientation needed for the FE simulations an implicit coordinate system similar to the one by Chandran et al. (2017) was introduced.

Initially four points were placed on the surface of the femoral head by approaching and intersecting a plane in four different directions. An initial sphere was then fitted based on the four points. The center of the so-found sphere was used to define a polar coordinate system, which was the basis to create a set of 200 rays pointing from the center to the surface of the sphere. The intersection of the 200 rays with the sphere surface were recorded and used to refine the fit of the sphere. This way the femoral head center and head radius were found. In a next step a series of progressively dilated spheres around the femoral head were intersected with the bone mask. The centers of mass of the consecutive intersections were computed and the linear regression of the so-found centers of mass resulted in a first crude estimate of the femoral neck axis. The initial estimate was refined by fitting a line to the centers of mass of the intersections between the bone mask and a series of disks perpendicular to the initial neck axis.

To standardize the proximal shaft axis detection without precise knowledge of the distal cut location, a two-step procedure was adopted. Approaching in infero-superior direction, the number of pixels was computed in each section, and the first “complete” section of the shaft was defined as the one with less than 5% variation in number of pixels with respect to the former. The centroids of the slices within 1cm above the first complete section were used to fit an initial shaft axis. A closest point

between the (generally not intersecting) femoral neck and shaft axes was computed and used to correct both axes.

The neck and proximal shaft axes were used to estimate the location of the lesser trochanter (LT). The LT surface was then segmented based on a measure of local curvature of the slice-wise contoured bone and the peak of the LT was placed at the slice with the largest curvature. The section of the shaft extending distally from the base of the LT over 15 mm (scaled with the head radius to account for inter-subject variability) was used as standard location for the estimation of the proximal femoral shaft axis. Centroids of each slice in this section were computed. In addition, the slice centroid of the section with the smallest eccentricity above the LT was detected. Two candidate guesses for the shaft axis were defined based on this information. The first guess was based on a linear regression of the computed slice centroids. The second guess was based on a RANSAC fit (Chandran et al., 2017). Based on the assumption that the initial estimate of the shaft axis was reasonably good, the candidate closer to the initial guess was kept. In cases where the distal length was not sufficient to adopt the described procedure, the initial shaft axis was kept or replaced by the axis defined by the most distal slice centroid and the centroid of the section with the smallest eccentricity above the LT.

The closest point between the neck axis and the so-found shaft axis were computed again. The so-obtained closest point in conjunction with the femoral head center and the slice center of the most distal slice were used to correct the femoral neck and shaft axis. The definition of the implicit coordinate system can thus be reduced to three points (head center, neck-shaft intersection point and most distal slice center).

A formal validation of this procedure was difficult to obtain and could thus not be performed. However, we tested the procedure on about 2600 3D DXA images and around 200 CT images and it worked reliably as assessed by visual control.

1.2.2 Stance configuration

Femoral strength is mostly computed in a configuration mimicking a side fall. We modeled also a more physiologic stance configuration like in Dall'Ara et al. (2013).

The mask of the bone was transformed rigidly so that the proximal shaft axis formed an angle of 20° with the vertical axis. An embedding cap was then applied on the femoral head with a depth of three to four voxels. The femur was cut at a distance corresponding to one head radius distal to the lesser trochanter. All nodes on the distal cut surface were fully constrained. A vertical displacement was applied to a node at the center of the head, which was kinematically coupled to the top surface of the embedding cap (Panyasantisuk et al., 2018). Supplementary Figure S2 shows the stance configuration with the boundary conditions.

Vertical displacements and reaction forces acting on the driving node in the femoral head center were recorded. In opposition to the fall load case a clear maximum in the force-displacement characteristic was reached in a large part of the cases in stance configuration. In these cases, strength was defined as the reaction force at the peak of the force-displacement curve. Otherwise, strength was defined as the reaction force when a displacement corresponding to 6% of the femoral head radius was reached.

After a first run where meshes based on QCT and 3D DXA images were treated independently, two different corrections for BMC differences between QCT and 3D DXA were introduced as for the side

fall condition. A collective correction based on the regression equation between the BMC values of 3D DXA-based and the QCT-based meshes, and an individual correction where the BMC of each 3D DXA-based mesh was adjusted to the corresponding QCT-based mesh

As for the side fall configuration the spatial distribution of damage (3D damage maps) was extracted for the QCT-based simulations and the individually corrected 3D DXA-based simulations, and an average damage distribution was computed.

The results of the simulations in stance configuration are shown in Supplementary Figure S4, Supplementary Figure S5 and Supplementary Table S2.

1.2.3 Constitutive model

The material model used for the FE simulations performed in this paper is an isotropic formulation of the model developed by Schwiedrzik and Zysset (2013) with yielding defined by the criterion described in Schwiedrzik et al. (2013).

The elastic stiffness tensor is given by

$$\mathbb{S}(\rho) = f(\rho)\left(\lambda_0\,\mathbf{I}\otimes\mathbf{I} + 2\mu_0\mathbf{I}\,\underline{\overline{\otimes}}\,\mathbf{I}\right)$$

Where $\lambda_0$and μ_0 are the Lamé constants with the positive-definiteness condition

$$3\lambda_0 + 2\mu_0 > 0 \qquad \mu_0 > 0$$

Accordingly, the compliance tensor is given by

$$\mathbb{E}(\rho) = \frac{1}{f(\rho)}\left(\frac{-\upsilon_0}{\epsilon_0}\mathbf{I}\otimes\mathbf{I} + \frac{(1+\ \upsilon_0)}{\varepsilon_0}\mathbf{I}\,\underline{\overline{\otimes}}\,\mathbf{I}\right)$$

Where ϵ_0 is the reference Young's modulus and υ_0 the Poisson ratio. The relationship between the Lamé constants and the engineering constants is given by:

$$\lambda_0 = \frac{\epsilon_0\upsilon_0}{(1+\upsilon_0)(1-2\upsilon_0)} \qquad \mu_0 = \frac{\epsilon_0}{2(1+\upsilon_0)}$$

$$\epsilon_0 = \frac{(3\lambda_0 + 2\mu_0)\mu_0}{\lambda_0 + \mu_0} \qquad \upsilon_0 = \frac{\lambda_0}{2(\lambda_0 + \mu_0)}$$

The function f(ρ) is the so-called tissue function introduced by Dall'Ara et al. (2013).

$$\begin{cases} f(\rho, E_{max}, \epsilon_0, k) = \rho^k & ,for\ \rho \leq 0.5 \\ f(\rho, E_{max}, \epsilon_0, k) = \ \rho^k + \dfrac{E_{max} - \varepsilon_0}{\varepsilon_0}\left(\dfrac{\rho - 0.5}{0.5}\right)^{2k} & ,for\ \rho\ > 0.5 \end{cases}$$

The tissue function modifies the power law for trabecular bone typically represented by low density elements, so that realistic stiffness values are obtained for high density elements that typically contain a large fraction of cortical bone. The piecewise relationship is illustrated in Supplementary Figure S3.

The quadric yield criterion (Schwiedrzik et al., 2013) coupled with a perfectly plastic post-yield behavior is given by:

$$\mathrm{Y}(\mathbf{S}) = \sqrt{\mathbf{S}:\mathbb{F}\mathbf{S}} + \mathbf{F}:\mathbf{S} - 1$$

The origin, orientation and shape of the criterion are defined by the fourth-order tensor $\mathbb{F}$ and second order tensor **F.**

$$\mathbb{F}(\rho) = \frac{1}{f^2(\rho)}\left(\frac{-\zeta_0}{4}\left(\frac{1}{\sigma_0^+} + \frac{1}{\sigma_0^-}\right)^2 \mathbf{I} \otimes \mathbf{I} + \frac{1+\zeta_0}{4}\left(\frac{1}{\sigma_0^+} + \frac{1}{\sigma_0^-}\right)^2 \mathbf{I} \,\overline{\underline{\otimes}}\, \mathbf{I}\right)$$

$$\mathbf{F}(\rho) = \frac{1}{f(\rho)}\frac{1}{2}\left(\frac{1}{\sigma_0^+} - \frac{1}{\sigma_0^-}\right)\mathbf{I}$$

Where σ_0^+ and σ_0^- are the tensile and compressive yield stresses. In the isotropic case, yielding through shearing is given by τ_0:

$$\tau_0 = \left(\frac{1}{\sigma_0^+} + \frac{1}{\sigma_0^-}\right)^{-1}\sqrt{\frac{2}{1+\zeta_0}}$$

Where ζ_0 is the so-called interaction parameter (Schwiedrzik et al., 2013).

The total Green-Lagrange strain **E** is decomposed in its elastic $\mathbf{E}^{\mathbf{e}}$ and plastic $\mathbf{E}^{\boldsymbol{p}}$ parts.

$$\mathbf{E} = \mathbf{E}^{\mathbf{e}} + \mathbf{E}^{\mathbf{p}}$$

Stiffness is isotropically reduced by a damage variable $D(\kappa)$ resulting in the following equation for the stress:

$$\boldsymbol{S} = \left(1 - D(\kappa)\right)\mathbb{S}(\mathbf{E} - \mathbf{E}^{\mathbf{p}})$$

The isotropic damage variable $\boldsymbol{D}(\boldsymbol{\kappa})$ is driven by the cumulated plastic strain $\boldsymbol{\kappa}$:

$$\boldsymbol{D}(\boldsymbol{\kappa}) = \boldsymbol{D}_{\boldsymbol{c}}(\mathbf{1} - \boldsymbol{e}^{-\boldsymbol{k}_{\boldsymbol{p}}\boldsymbol{\kappa}})$$

Where $\boldsymbol{\kappa}$ is given by:

$$\boldsymbol{\kappa} = \int_{\mathbf{0}}^{\boldsymbol{t}} \left\|\dot{\mathbf{E}}^{\mathbf{p}}\right\| \boldsymbol{d\tau}$$

The numerical values of the constants used are given in Supplementary Table S1.

1.2.4 BMD to BV/TV conversion

BMD values have been converted to µCT equivalent bone volume fractions (BV/TV) according to the relationship established by Dall'Ara et al. (2013).

$$\begin{cases} \rho = \mu CT_{BV/TV}[\%] = 0.093 \cdot QCT_{BMD} \dfrac{mmHg}{cm^3} + 1.077 \quad , & for -100 < QCT_{BMD} \leq 1064 \\ \rho = \mu CT_{BV/TV}[\%] = 100 \quad , & for\ 1064 < QCT_{BMD} \leq 1400 \end{cases}$$

2 Supplementary Figures

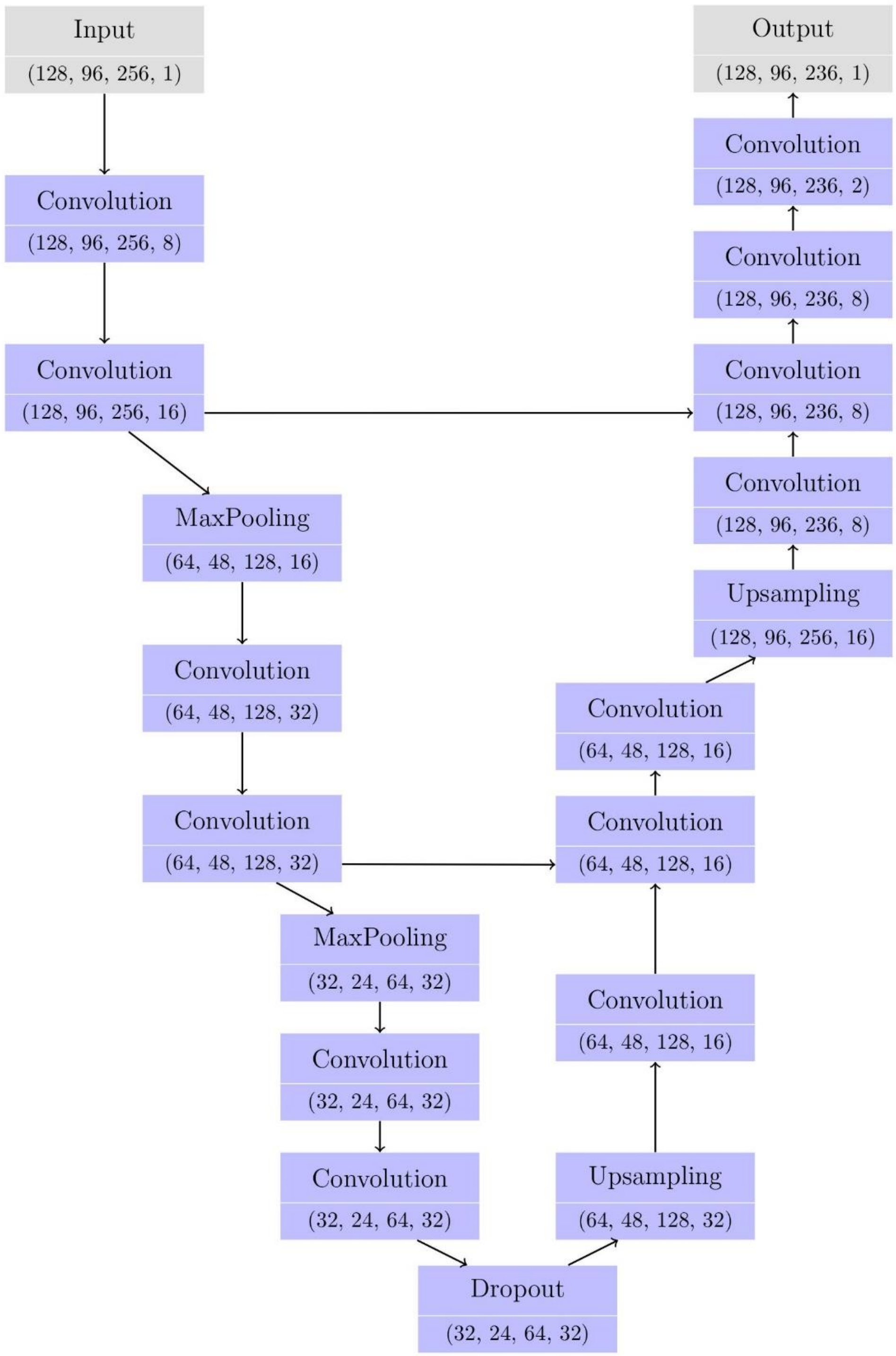

Supplementary Figure S1: Network architecture of the QCT segmentation model.

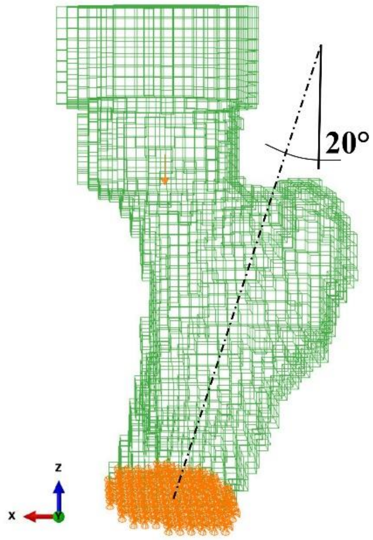

Supplementary Figure S2: Stance configuration: The axis of the proximal shaft is in an angle of 20° with the vertical. The nodes on the distal cut surface are fully constrained. A displacement is applied to a node in the center of the head, which is rigidly coupled to the top surface of the embedding cap on the femoral head.

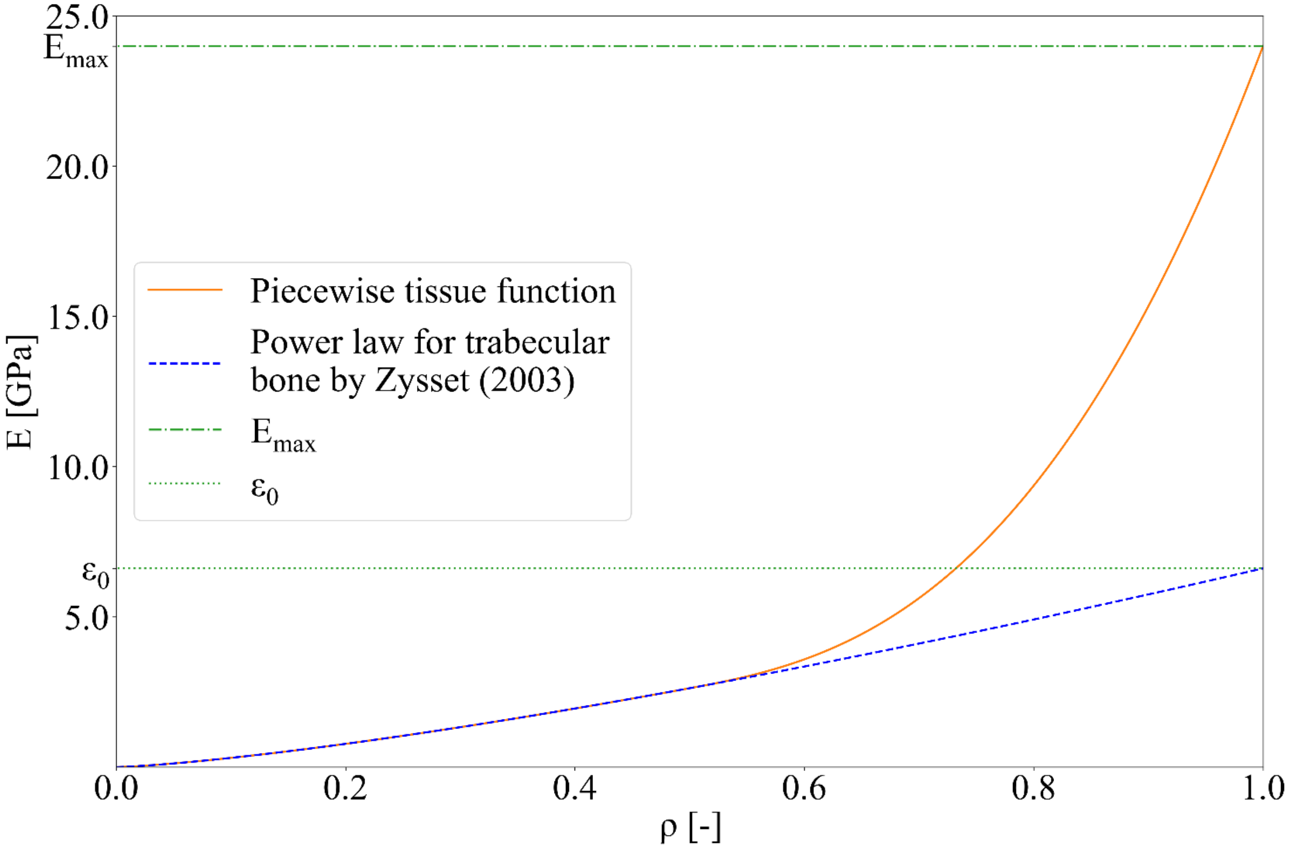

Supplementary Figure S3: Piecewise tissue function $\mathbf{f}(\boldsymbol{\rho})$. A Young's modulus of 24 GPa is obtained for idealized pore-less bone (BV/TV = 1).

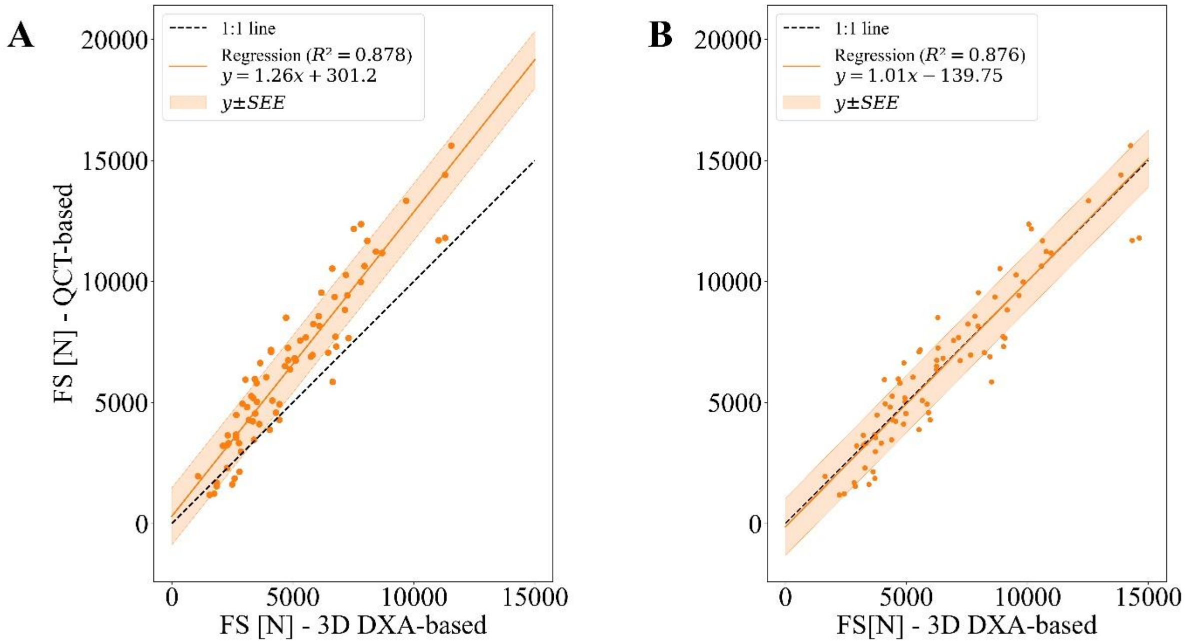

Supplementary Figure S4: Linear regression between 3D DXA-based FE results and QCT-based FE results for the stance configuration. A) 3D DXA images processed independently of QCT images. B) BMC of 3D DXA-based meshes adjusted to BMC of QCT-based meshes using the linear regression equation between QCT-based and 3D DXA-based BMC values (collectively corrected simulations). The regression line sticks closely to the bisecting line (1:1 line). As it can be seen in Supplementary Table S2 the slope is not significantly different from 1 and the intercept is not significantly different from 0 at a 95% confidence level for this case.

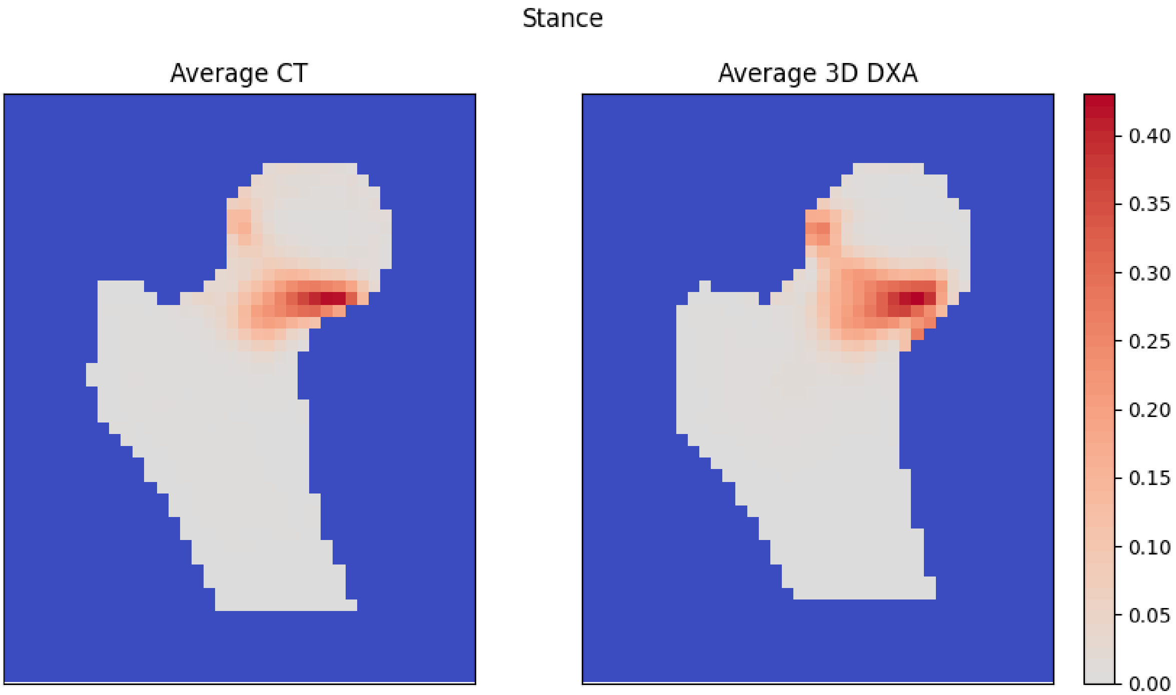

Supplementary Figure S5: Average damage maps for QCT and 3D DXA in stance configuration. The zones of high damage, which are the zones where fracture initiation can be expected, are matching.

3 Supplementary Tables

Supplementary Table S1: Material constants for bone constitutive model. Elasticity and yield constants have been fitted to match the experimental results of (Rincón-Kohli and Zysset, 2009). The constants $\boldsymbol{D_c}$ and $\boldsymbol{k_p}$ used in the damage formulation were adjusted according to the experimental findings of Wolfram et al. (2011).

σ_0^+ [MPa]	σ_0^- [MPa]	τ_0 [MPa]	ζ_0 [-]	ε_0 [MPa]	υ_0 [-]	k [-]	E_{max} [MPa]	D_c [-]	k_p [-]
54.795	72.877	39.962	0.225	6614.07	0.2462	1.333	24’000	0.85	8.0075

Supplementary Table S2: Linear regression results, error measures and concordance for stance configuration

	QCT vs. uncorrected 3D DXA-based simulations	QCT vs. collectively corrected 3D DXA-based simulations	QCT vs. individually corrected 3D DXA-based simulations	QCT-based FE simulation vs. DXA-based FN aBMD
**N**	77			
**R^2**	0.878	0.876	0.908	0.831
**Slope (95% CI) [-]**	1.26 ± 0.11 [-]	1.01 ± 0.09 [-]	1.01 ± 0.08 [-]	17649 ± 1829 $[\frac{N}{\frac{g}{cm^2}}]$
**Intercept (95% CI) [N]**	301.2 ± 597.2	-139.8 ± 636.0	-128.0 ± 538.0	-5514 ± 1287
**SEE [N]**	1176	1184	1019	1383
**Avg. predictor strength (3D DXA) [N]**	4955 ± 2476	6575 ± 3065	6609 ± 3142	N/A
**Avg. predicted Strength (QCT) [N]**	6530 ± 3322			
**CV of SEE [%]**	18.0	18.1	15.6	21.1
**MAE [N]**	1698	950	775	N/A
**CV of MAE [%]**	26.0	14.6	11.9	N/A
**Concordance correlation coefficient [-]**	0.796	0.924	0.947	N/A

4 References

Chandran, V., Reyes, M., Zysset, P., 2017. A novel registration-based methodology for prediction of trabecular bone fabric from clinical QCT: A comprehensive analysis. PLoS ONE 12, e0187874. https://doi.org/10.1371/journal.pone.0187874

Dall'Ara, E., Luisier, B., Schmidt, R., Kainberger, F., Zysset, P., Pahr, D., 2013. A nonlinear QCT-based finite element model validation study for the human femur tested in two configurations in vitro. Bone 52, 27–38. https://doi.org/10.1016/j.bone.2012.09.006

Kingma, D.P., Ba, J., 2017. Adam: A Method for Stochastic Optimization. https://doi.org/10.48550/arXiv.1412.6980

Panyasantisuk, J., Dall'Ara, E., Pretterklieber, M., Pahr, D.H., Zysset, P.K., 2018. Mapping anisotropy improves QCT-based finite element estimation of hip strength in pooled stance and side-fall load configurations. Medical Engineering & Physics 59, 36–42. https://doi.org/10.1016/j.medengphy.2018.06.004

Rincón-Kohli, L., Zysset, P.K., 2009. Multi-axial mechanical properties of human trabecular bone. Biomech Model Mechanobiol 8, 195–208. https://doi.org/10.1007/s10237-008-0128-z

Ronneberger, O., Fischer, P., Brox, T., 2015. U-Net: Convolutional Networks for Biomedical Image Segmentation, in: Navab, N., Hornegger, J., Wells, W.M., Frangi, A.F. (Eds.), Medical Image Computing and Computer-Assisted Intervention – MICCAI 2015, Lecture Notes in Computer Science. Springer International Publishing, Cham, pp. 234–241. https://doi.org/10.1007/978-3-319-24574-4_28

Schwiedrzik, J.J., Wolfram, U., Zysset, P.K., 2013. A generalized anisotropic quadric yield criterion and its application to bone tissue at multiple length scales. Biomech Model Mechanobiol 12, 1155–1168. https://doi.org/10.1007/s10237-013-0472-5

Schwiedrzik, J.J., Zysset, P.K., 2013. An anisotropic elastic-viscoplastic damage model for bone tissue. Biomech Model Mechanobiol 12, 201–213. https://doi.org/10.1007/s10237-012-0392-9

Wolfram, U., Wilke, H.-J., Zysset, P.K., 2011. Damage accumulation in vertebral trabecular bone depends on loading mode and direction. Journal of Biomechanics 44, 1164–1169. https://doi.org/10.1016/j.jbiomech.2011.01.018
